# Spatiotemporal control of interferon-induced JAK/STAT signalling and gene transcription by the retromer complex

**DOI:** 10.1038/ncomms13476

**Published:** 2016-12-05

**Authors:** Daniela Chmiest, Nanaocha Sharma, Natacha Zanin, Christine Viaris de Lesegno, Massiullah Shafaq-Zadah, Vonick Sibut, Florent Dingli, Philippe Hupé, Stephan Wilmes, Jacob Piehler, Damarys Loew, Ludger Johannes, Gideon Schreiber, Christophe Lamaze

**Affiliations:** 1 Membrane Dynamics and Mechanics of Intracellular Signaling Laboratory, Institut Curie–Centre de Recherche, PSL Research University, 26 rue d'Ulm, F-75248 Paris, France; 2 Institut National de la Santé et de la Recherche Médicale (INSERM) U1143, 75005 Paris, France; 3 Centre National de la Recherche Scientifique (CNRS), UMR 3666, 75005 Paris, France; 4 Department of Biological Chemistry, Weizmann Institute of Science, Rehovot 76100, Israel; 5 Endocytic Trafficking and Intracellular Delivery Laboratory, Institut Curie–Centre de Recherche, PSL Research University, F-75248 Paris, France; 6 Bioinformatics and Computational Systems Biology of Cancer, Institut Curie–Centre de Recherche, PSL Research University, F-75248 Paris, France; 7 INSERM U900, 75005 Paris, France; 8 Mines Paris-Tech, F-75272 Paris, France; 9 Proteomics and Mass Spectrometry Laboratory, Institut Curie–Centre de Recherche, PSL Research University, F-75248 Paris, France; 10 CNRS UMR144, 75005 Paris, France; 11 Division of Biophysics, Department of Biology, University of Osnabrück, 49074 Osnabrück, Germany

## Abstract

Type-I interferons (IFNs) play a key role in the immune defences against viral and bacterial infections, and in cancer immunosurveillance. We have established that clathrin-dependent endocytosis of the type-I interferon (IFN-α/β) receptor (IFNAR) is required for JAK/STAT signalling. Here we show that the internalized IFNAR1 and IFNAR2 subunits of the IFNAR complex are differentially sorted by the retromer at the early endosome. Binding of the retromer VPS35 subunit to IFNAR2 results in IFNAR2 recycling to the plasma membrane, whereas IFNAR1 is sorted to the lysosome for degradation. Depletion of VPS35 leads to abnormally prolonged residency and association of the IFNAR subunits at the early endosome, resulting in increased activation of STAT1- and IFN-dependent gene transcription. These experimental data establish the retromer complex as a key spatiotemporal regulator of IFNAR endosomal sorting and a new factor in type-I IFN-induced JAK/STAT signalling and gene transcription.

## Abstract (web summary)

Endocytosis of the type I interferon receptor (IFNAR) is required for JAK/STAT signalling. Here the authors show that the internalized IFNAR1 and IFNAR2 subunits are differentially sorted by the retromer complex at the early endosome and this controls JAK/STAT signalling and gene transcription.

## Graphical abstract

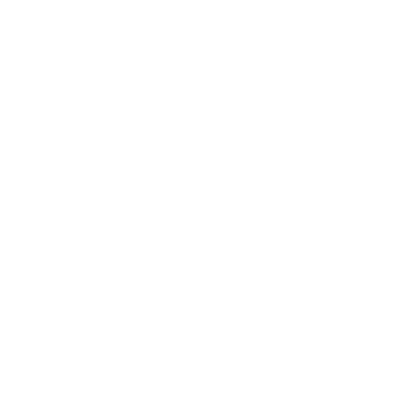

Type-I interferons (IFN-α/β) are key cytokines for cellular responses in innate and acquired immunity to diseases including cancer and infection^1^. The type-I IFNs activate the canonical Janus kinase/signal transducers and activators of transcription (JAK/STAT) signalling pathway, which relies on ubiquitously expressed type I IFN-α/β receptors (IFNAR), Janus family kinases (JAK1 and TYK2), and STAT1 and STAT2. IFN-α/β binding results in IFNAR1 and IFNAR2 subunits rearrangement and dimerization, followed by auto- and transphosphorylation and activation of TYK2 and JAK1, which are respectively pre-associated with IFNAR1 and IFNAR2 (refs 1, 2). JAK-mediated IFNAR phosphorylation leads to the recruitment and activation of cytoplasmic STAT1 and STAT2, which in association with IFN-regulatory factor 9, are translocated to the nucleus as a trimolecular complex called IFN-stimulated gene (ISG) factor 3 that binds DNA to initiate the transcription of ISGs.

As many as 17 different but related type-I IFNs elicit numerous and complex activities through binding to the same IFNAR receptor complex, raising the question of the molecular mechanisms that control the selectivity of type-I IFN signalling. Several studies have established various regulatory mechanisms at the level of gene transcription, epigenetics or signalling cross-talks^3^,^4^,^5^. Endocytosis has long been viewed as a simple means to control receptor signalling by down-modulation of receptor numbers at the plasma membrane. Pioneering studies on the epidermal growth factor receptor have challenged this passive role of endocytosis^6^ and led to a new paradigm where the endocytic network is directly connected to the control of receptor signalling^7^. If increasing evidence suggests that endosomes may function as signalling platforms, the challenge today is to identify the molecular machinery that controls the endocytosis signalling nexus^8^. This particular aspect of receptor signalling regulation by membrane trafficking has received little attention for IFNAR and JAK/STAT signalling^9^,^10^. Nevertheless, we previously established that IFNAR endocytosis through clathrin-coated pits was required for the activation of JAK/STAT signalling and the antiviral and antiproliferative activities of type-I IFNs^11^.

In this study, we aimed to elucidate how the delivery of IFNAR to the endosomal network may play a role in the control of JAK/STAT signalling induced by IFN-α/β. We consequently uncover a new role for the retromer complex in JAK/STAT signalling termination. We found that the retromer subunit vacuolar protein sorting-associated protein 35 (VPS35) binds IFNAR2 and thereby controls the spatiotemporal dissociation of the IFNAR1 and IFNAR2 subunits of the IFNAR complex at the early endosome. This interaction is critical for the proper regulation of JAK/STAT signalling and gene transcription induced by IFN-α/β.

## Results

### Different sorting of IFNAR1 and IFNAR2 at the early endosome

**Figure 1:**
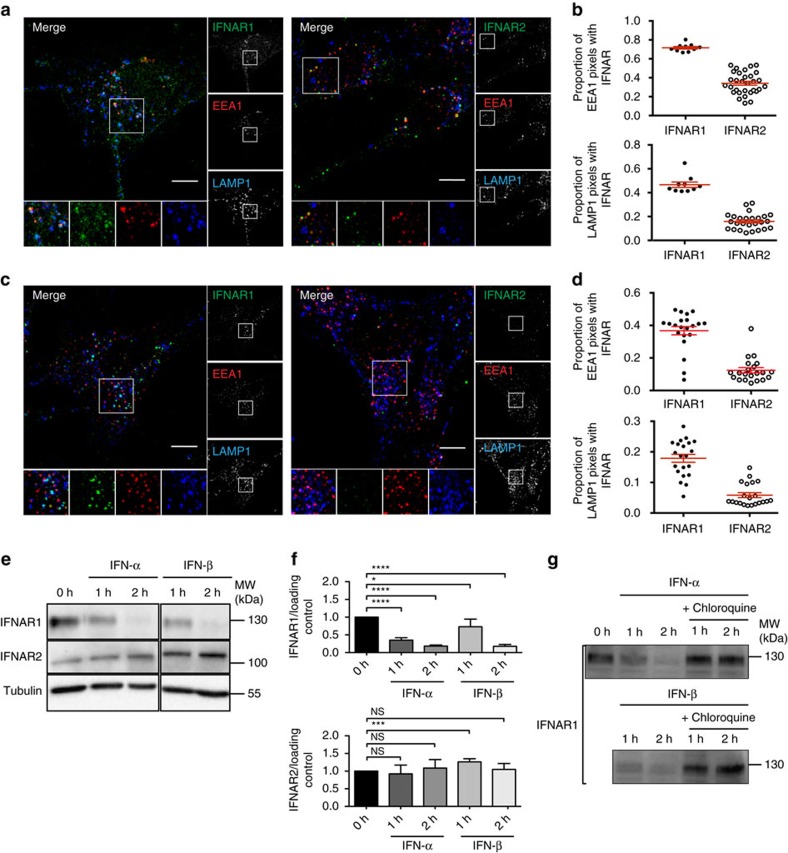
Intracellular localization of IFNAR1 and IFNAR2 subunits after IFNAR endocytosis. (**a**) Immunofluorescent labelling of endocytosed IFNAR1 and IFNAR2 subunits on 20 min IFN-α stimulation in RPE1 cells. Following fixation, cells were co-labelled for EEA1 and LAMP1, and analysed by confocal microscopy. (**b**) Quantification of co-localization in **a** expressed as the Manders' coefficient, indicating the proportion of EEA1 pixels (upper panel) or LAMP1 pixels (lower panel) containing IFNAR1 and IFNAR2 pixels. (**c**) Immunofluorecent labelling of endocytosed IFNAR1 and IFNAR2 subunits on 60 min IFN-α stimulation. Following fixation, cells were co-labelled for EEA1 and LAMP1, and analysed by confocal microscopy. (**d**) Quantification of co-localization in **c** expressed as the Manders' coefficient, indicating the proportion of EEA1 pixels (upper panel) or LAMP1 pixels (lower panel) containing IFNAR1 and IFNAR2 pixels. (**e**) Immunonoblot analysis of total levels of IFNAR1 and IFNAR2 subunits in RPE1 cells on IFN stimulation. Cells were pretreated with cycloheximide and stimulated with IFN-α or IFN-β at 37 °C for the indicated times. (**f**) Quantification of experiments performed in **e**: IFNAR1 or IFNAR2 level was normalized to loading control level (tubulin or CHC, clathrin heavy chain) and the ratio IFNAR/loading control was calculated for each condition. (**g**) Immunoblot analysis of total IFNAR1 level in RPE1 cells on IFN stimulation. Cells were pretreated with cycloheximide and chloroquine, followed by IFN-α or IFN-β at 37 °C for the indicated times. Scale bars, 10 μm. Reproducibility of experiments: (**a**,**b**) (upper panel: *n*=10 and 30 cells, respectively, and lower panel: *n*=10 and 26 cells, respectively, for each condition) and **c**,**d** (*n*=21 cells per condition) show representative data of three independent experiments; **e** shows representative data and **f** shows quantification of IFNAR1 of *n*=8, *n*=4, *n*=8, *n*=3, *n*=6 independent experiments, respectively, for each time point, and of IFNAR2 of *n*=8, *n*=4, *n*=6, *n*=3, *n*=4 independent experiments, respectively. Statistical analysis with Mann–Whitney test was performed. *P<0.05, ***P<0.001 and ****P<0.0001; NS, nonsignificant. **g** shows representative data for three independent experiments. Graphs **b**,**d** and **f** show mean value±s.e.m.

To address the role of endosomal sorting in JAK/STAT signalling, we first characterized the intracellular dynamics of the IFNAR complex subunits in RPE1 cells. Live-cell, single-molecule total internal reflection fluorescence imaging of bound fluorescently labelled engineered IFNs revealed a high cell surface expression of endogenous IFNAR1 and IFNAR2 levels in RPE1 cells (Supplementary Fig. 1a,b). Compared with HeLa cells, RPE1 express ∼1.5 × higher IFNAR1 levels (RPE1: 0.58±0.08 IFNAR1 per μm^2^; HeLa: 0.41±0.10 IFNAR1 per μm^2^) and ∼2 × higher IFNAR2 levels (RPE1: 0.72±0.10 IFNAR2 per μm^2^; HeLa: 0.34±0.08 IFNAR2 per μm^2^), and thus enable the analysis of IFNAR trafficking with standard immunofluorescent assays. After 20 min of IFNAR endocytosis in the presence of IFN-α, most endosomal structures positive for EEA1, a *bona fide* marker of the early endosome, also contained IFNAR1 and IFNAR2 subunits (Fig. 1a,b). We also found IFNAR1 subunits in the LAMP1-positive lysosomal compartments in agreement with previously reported ubiquitin-dependent degradation of IFNAR1 (refs 12, 13). At 60 min of uptake, a time classically required for cargo degradation, IFNAR1 subunits were still found in lysosomes (Fig. 1c,d). However, the co-localization between LAMP1 and IFNAR1 was markedly decreased as compared with 20 min, most probably as a result of IFNAR1 lysosomal degradation. In contrast to IFNAR1, very few LAMP1-positive structures contained IFNAR2 after 20 and 60 min of uptake (Fig. 1a–d). We confirmed these data by measuring the total levels of IFNAR1 and IFNAR2 subunits by western blot (c) analysis in the presence of cycloheximide to block protein neosynthesis (Fig. 1e,f). Under these conditions, we found that the total amount of IFNAR1 present in RPE1 cells was significantly decreased after 1 h IFN-α or IFN-β stimulation. After 2 h of IFN-α or IFN-β stimulation, IFNAR1 was no longer detectable in RPE1 cells due to its degradation in lysosomes. Indeed, addition of the lysosomotropic agent chloroquine that blocks lysosomal acidification prevented IFNAR1 degradation (Fig. 1g). In contrast, IFNAR2 was not degraded on IFN stimulation. These results therefore show that IFNAR1 but not IFNAR2 undergoes ligand-dependent ubiquitination and degradation in agreement with previous reports^14^. They also suggest that after endocytosis, the IFNAR1 and IFNAR2 subunits follow different intracellular pathways.

**Figure 2:**
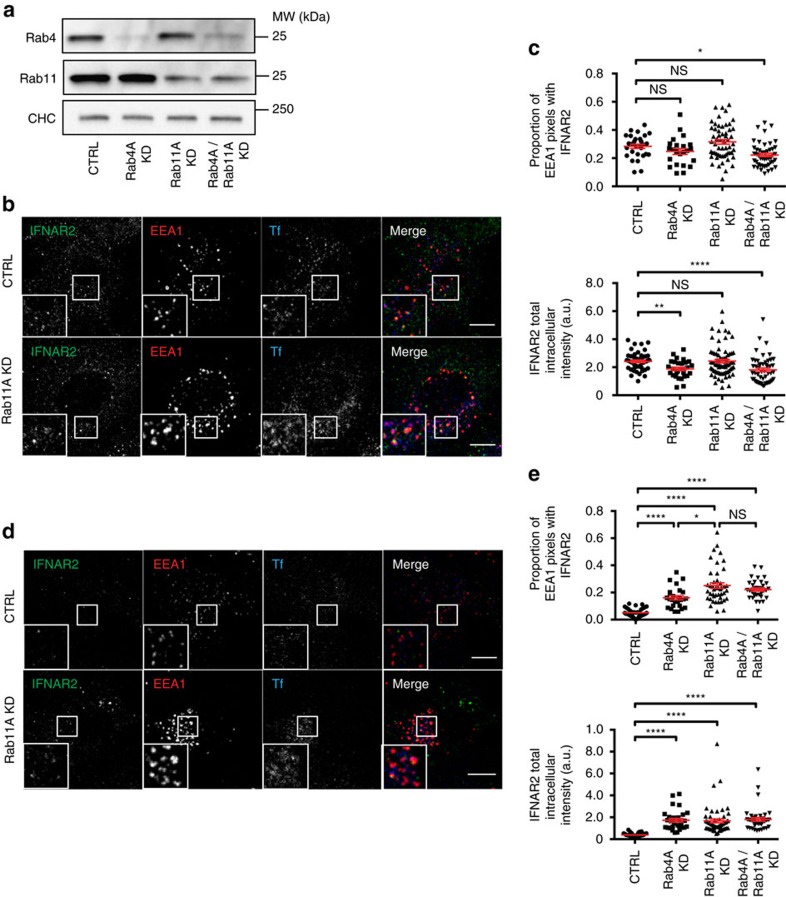
Rab11A and Rab4A control IFNAR2 recycling from the early endosome to the plasma membrane. (**a**) Immunoblot for Rab4A, Rab11A and Rab4A/Rab11A knockdown efficiencies measured after 72 h of siRNA transfection. Clathrin heavy chain (CHC) was used as a loading control. (**b**) Uptake of Tf-Alexa647 conjugate and IFNAR2 subunit in control (CTRL) and Rab4A/Rab11A-depleted RPE1 cells on 20 min IFN-α stimulation. Following fixation, cells were co-labelled for EEA1 and analysed by confocal microscopy. (**c**) Quantification of co-localization in **b** expressed as the Manders' coefficient, indicating the proportion of EEA1 pixels containing IFNAR2 pixels (upper panel); level of total intracellular intensity of endocytosed IFNAR2 in CTRL, Rab4A-, Rab11A- or Rab4A/Rab11A-depleted RPE1 cells (lower panel). (**d**) Uptake of Tf-Alexa647 conjugate and IFNAR2 subunit in control (CTRL) and Rab4A/Rab11A-depleted RPE1 cells on 60 min IFN-α stimulation. Following fixation, cells were co-labelled for EEA1 and LAMP1, and analysed by confocal microscopy. (**e**) Quantification of co-localization in **d** expressed as the Manders' coefficient, indicating the proportion of EEA1 pixels containing IFNAR2 pixels (upper panel); level of total intracellular intensity of endocytosed IFNAR2 in CTRL, Rab4A-, Rab11A- or Rab4A/Rab11A-depleted RPE1 cells (lower panel). Scale bars, 10 μm. Reproducibility of experiments: (**a**,**b**,**c**) (upper panel: *n*=29, *n*=23, *n*=57, *n*=47 and lower panel: *n*=48, *n*=28, *n*=73, *n*=51 cells, respectively, for each condition), **d** and **e** (upper panel: *n*=37, *n*=26, *n*=44, *n*=34 and lower panel: *n*=40, *n*=30, *n*=57, *n*=41 cells, respectively, for each condition) show representative data for three independent experiments. Graphs **c** and **e** show mean value±s.e.m.; statistical analysis with Kruskal–Wallis test was performed. *P<0.05, **P<0.01 and ****P<0.0001; NS, nonsignificant.

We therefore analysed the possibility that IFNAR2 may recycle to the plasma membrane after being sorted at the early endosome. To do so, we followed IFNAR2 trafficking in RPE1 cells depleted or not from Rab11A and/or Rab4A, two small GTPases that are classically involved in the selective control of cargo recycling to the plasma membrane (Fig. 2a)^15^. After 20 min of IFNAR endocytosis, we found that IFNAR2 was present in EEA1-positive endosomes to a similar extent in control and Rab11A-depleted cells. A similar phenotype was observed in Rab4A- and Rab4A/Rab11A-depleted cells (Fig. 2b,c). Accordingly, the overall intensity of IFNAR2 intracellular staining was identical between control and Rab4A- or Rab11A-depleted cells (Fig. 2c).

After 60 min of IFNAR uptake however, IFNAR2 was retained in EEA1 endosomes in Rab11A-depleted cells, whereas it was no longer present in control cells (Fig. 2d,e). Accordingly, we found that the overall intensity of IFNAR2 intracellular staining was significantly increased in Rab11A-depleted cells (Fig. 2e). There was also a significant contribution of Rab4A in the retention of IFNAR2 in early endosomes (Fig. 2e). In agreement with published results, fluorescently labelled transferrin (Tf) that binds to the Tf receptor, a *bona fide* marker of the recycling pathway, was accumulated in Rab11A-depleted cells (Fig. 2d). Depletion of both Rab4A and Rab11A did not further increase the level of IFNAR2 retention in the endosome (Fig. 2e), nor did it change IFNAR1 intracellular trafficking (Supplementary Fig. 1c,d). Altogether, these experimental data indicate that after IFNAR endocytosis and delivery to the early endosome, IFNAR2 but not IFNAR1 recycles back to the plasma membrane in a Rab4A- and Rab11A-regulated manner. These results are therefore in agreement with the lysosomal degradation of IFNAR1 after endocytosis and the recycling of IFNAR2 to the plasma membrane for further rounds of endocytosis.

### VPS35 of the retromer binds IFNAR2 at the early endosome

To gain more insight into the molecular machinery that is actively sorting the IFNAR complex at the early endosome, we performed proteomic analysis in RPE1 cells after stimulation or not with IFN-α or IFN-β for 10 min, a standard time that corresponds to the delivery of the endocytosed IFNAR to the early endosome. Endogenous IFNAR1 and IFNAR2 were immunoprecipitated in RPE1 cell lysates and analysed by mass spectrometry, to identify partners of the IFNAR subunits present at the early endosome. Our mass spectrometry analysis revealed the presence of expected partners of the IFNAR complex such as STAT1 (Supplementary Table 1). We also found several subunits of the retromer complex namely VPS26A, VPS29 and VPS35 associated with IFNAR2 (Supplementary Table 1). In the context of the ability of IFNAR2 to recycle from the early endosome to the plasma membrane, we therefore decided to further characterize the possible interaction of IFNAR2 with the retromer complex as suggested by the proteomics data.

**Figure 3:**
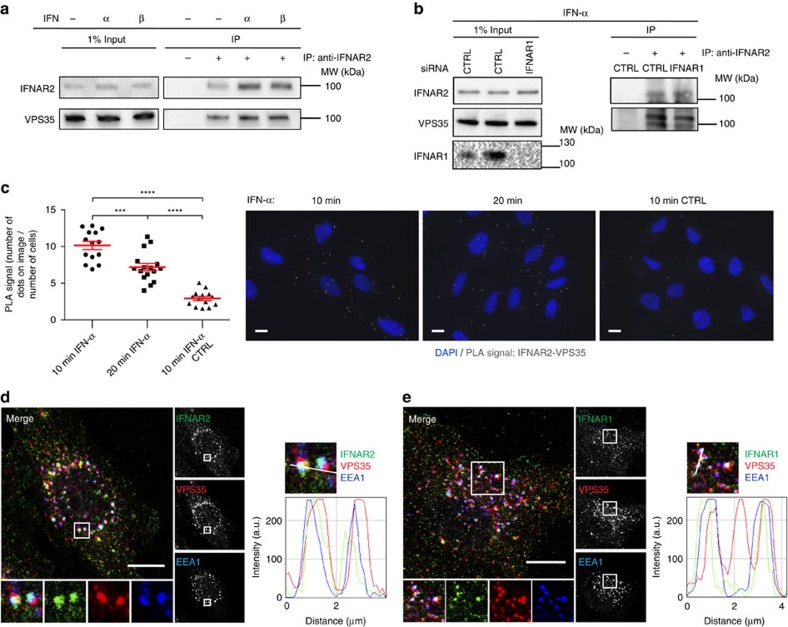
IFNAR interaction with VPS35. (**a**) Untransfected RPE1 cells were stimulated or not (−) with IFN-α or IFN-β as indicated. Cell lysates were incubated with anti-IFNAR2 antibody to immunoprecipitate (IP) endogenous IFNAR2 and reveal co-immunoprecipitated endogenous VPS35. (**b**) CTRL and IFNAR1-depleted RPE1 cells were stimulated for 10 min with IFN-α. Cell lysates were incubated with anti-IFNAR2 antibody to immunoprecipitate endogenous IFNAR2 and reveal co-immunoprecipitated endogenous VPS35. (**c**) Right, *in situ* PLA monitoring the interaction of endocytosed IFNAR2 with VPS35 after 10 min and 20 min IFN-α stimulation of RPE1 cells. Cells were analysed by epifluorescence microscopy. Control condition was performed in the absence of primary antibody against VPS35. Left, quantification from three independent experiments, each symbol on the graph represents one analysed field. (**d**) Left, immunofluorescent labelling of endocytosed IFNAR2 in RPE1 cells on 20 min IFN-α stimulation of RPE1 cells. Cells were fixed, co-labelled for VPS35 and EEA1, and analysed by confocal microscopy. Right, representative histogram of co-localization profile prepared with RGB profiler plugin of ImageJ. (**e**) Left, immunofluorescent labelling of endocytosed IFNAR1 in RPE1 cells on 20 min IFN-α stimulation. Cells were fixed, co-labelled for VPS35 and EEA1, and analysed by confocal microscopy. Right, representative histogram of colocalization profile prepared with RGB profiler plugin of ImageJ. Scale bars, 10 μm. Reproducibility of experiments: (**a** and **b**) show representative data of five and three independent experiments, respectively. (**c**) Left panel (*n*=14, *n*=16 and *n*=14 fields, respectively, for each condition) shows quantification of three independent experiments. Mean value±s.e.m.; statistical analysis with one-way analysis of variance and Sidak's multiple comparison test was performed. ***P<0.001 and ****P<0.0001NS, nonsignificant. (**c**) Right panels show representative data from three independent experiments. (**d**,**e**) Representative data for three independent experiments.

The retromer complex is a key element of the endosomal sorting machinery that has been mainly but not exclusively associated with the endosome-to-Golgi retrieval pathway^16^,^17^. The retromer is a pentameric complex consisting of two distinct subcomplexes. The VPS35/29/26 subcomplex forms a stable trimer that mediates cargo selection for endosomal sorting. The other subcomplex in mammalian cells is composed of sorting nexin heterodimers consisting of Snx1 or Snx2 with Snx5 or Snx6 (refs 16, 18, 19). Co-immunoprecipitation experiments of endogenous IFNAR2 in RPE1 cells confirmed the mass spectrometry analysis and showed an interaction between IFNAR2 and VPS35 at steady state. Stimulation of cells with IFN-α or IFN-β for 10 min led to a systematic but not statistically significant increase of VPS35–IFNAR2 interaction (Fig. 3a). We next analysed whether the interaction of VPS35 with IFNAR2 was mediated by IFNAR1, as IFN-α/β binding to the high-affinity IFNAR2 subunit allows the subsequent recruitment of IFNAR1 to the IFNAR complex at the plasma membrane^5^. In cells depleted from the IFNAR1 subunit however, we observed that IFNAR2 was still able to interact with VPS35 to the same extent than in control cells, indicating that VPS35 probably interacts with IFNAR2 directly (Fig. 3b). Conversely, co-immunoprecipitation experiments of endogenous IFNAR1 showed no interaction with VPS35 at steady state or on IFN stimulation (Supplementary Fig. 2a). We further analysed the interaction between IFNAR2 and VPS35 using the proximity ligation assay (PLA), which allows to directly visualize individual endogenous protein–protein interactions^20^. *In situ* PLA experiments confirmed the interaction between endogenous VPS35 and IFNAR2 (Fig. 3c). The IFNAR2–VPS35 interaction was maximal after 10 min of IFNAR uptake under IFN-α stimulation and decreased thereafter (20 min) most likely to be due to IFNAR subunits starting to leave the early endosome. Finally, we directly visualized by immunofluorescent confocal microscopy the intracellular localization of endocytosed IFNAR1 and IFNAR2 subunits with VPS35. In agreement with the abovementioned data, we found that IFNAR2 was present in early endosomes positive for VPS35 after 20 min uptake in the presence of IFN-α (Fig. 3d). In addition, IFNAR1 was also present in early endosomes and partially co-localized with VPS35 most probably indirectly through its association with IFNAR2 (Fig. 3e). We also examined the intracellular distribution of endogenous IFNAR1 and IFNAR2 subunits at steady state, that is, in the absence of IFN stimulation. In agreement with the tight interaction of VPS35 and VPS26 in the retromer complex, we found that IFNAR2 and both VPS26 and VPS35 co-localized in the same endosomes (Supplementary Fig. 2b,c). However, we could not detect a significant co-localization between IFNAR1 and VPS35 under the same experimental conditions (Supplementary Fig. 2d). These data confirm the results obtained by immunoprecipitation and indicate that VPS35 binds also to IFNAR2 at steady state in agreement with the recycling of IFNAR2 between the early endosome and the plasma membrane (Fig. 2a–d).

### The retromer complex is required for IFNAR endosomal sorting

**Figure 4:**
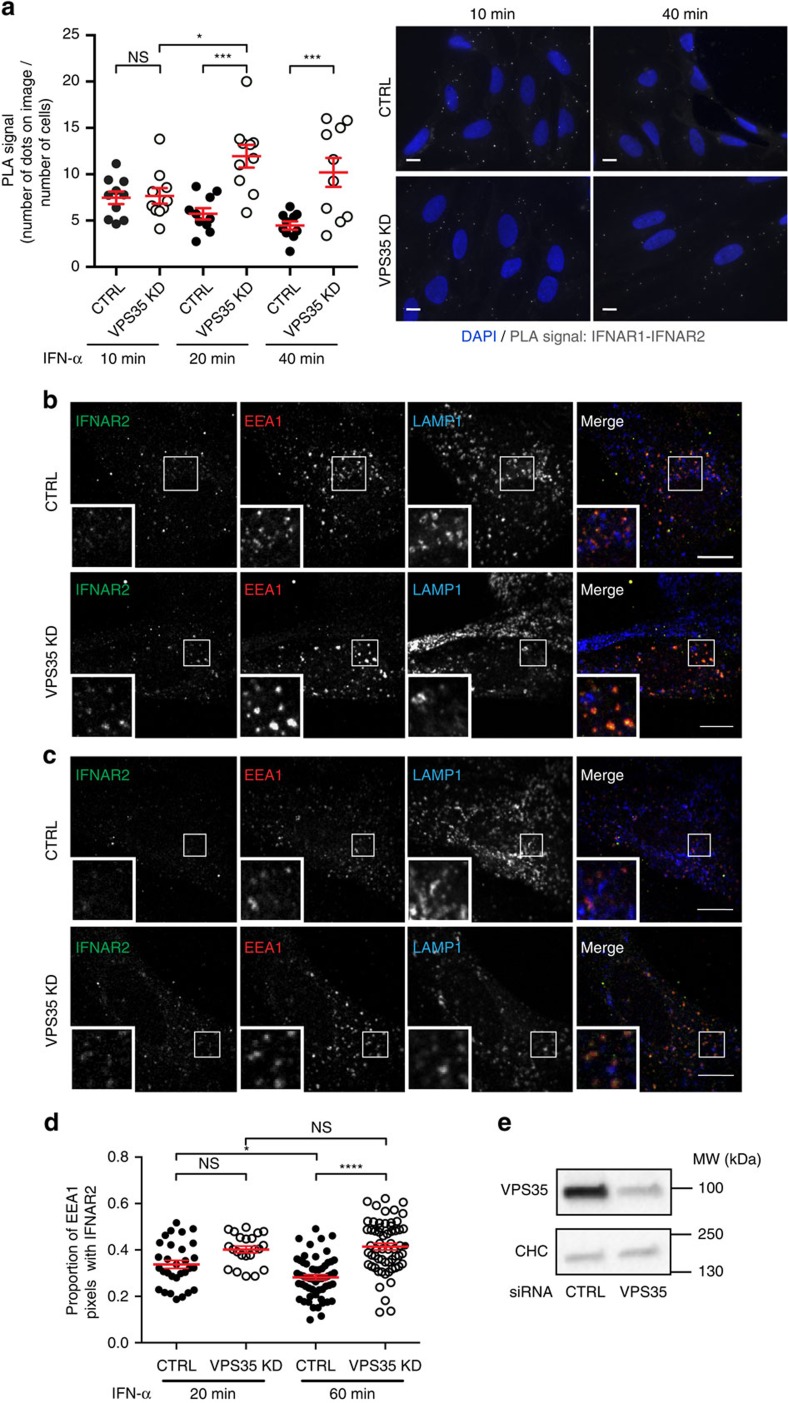
IFNAR2 intracellular trafficking is impaired in VPS35-depleted RPE1 cells. (**a**) Right, *in situ* PLA monitoring of IFNAR2 and IFNAR1 interaction after IFNAR endocytosis in CTRL and VPS35-depleted cells stimulated with IFN-α for the indicated times. Left, each symbol represents one analysed field. (**b**,**c**) Immunofluorescent labelling of endocytosed IFNAR2 in CTRL and VPS35-depleted cells after 20 min (**b**) and 60 min (**c**) of IFNAR uptake under IFN-α stimulation. Cells were fixed, co-labelled for EEA1 and LAMP1, and analysed by confocal microscopy. (**d**) Quantification of co-localization in **b**,**c** expressed as the Manders' coefficient, indicating the proportion of EEA1 pixels containing IFNAR2 pixels. (**e**) Immunoblot for VPS35 knockdown efficiency measured after 72 h of siRNA transfection. CHC was used as a loading control. Scale bars, 10 μm. Reproducibility of experiments: (**a**) left panel (each symbol represents one analyzed field, *n*=10 fields per condition) and right panels show representative data for three independent experiments. (**b**,**c**,**d**) (20 min: *n*=30, *n*=22 cells, respectively, per condition; 60 min *n*=57 cells, *n*=60 cells, respectively, per condition) and **e** show representative data for three independent experiments. Graphs **a** and **d** show mean value±s.e.m.; statistical analysis with one**-**way analysis of variance and Sidak's multiple comparison test was performed. *P<0.05, ***P<0.001 and ****P<0.0001; NS, nonsignificant.

We next analysed whether the retromer complex was actively involved in IFNAR endosomal sorting. We first analysed the dynamics of IFNAR1 and IFNAR2 interaction during the endocytic process. After 10 min IFNAR uptake, *in situ* PLA experiments revealed the same degree of association between IFNAR1 and IFNAR2 in both control and VPS35-depleted cells (Fig. 4a,e). At later times of endocytosis that correspond to the exit of the IFNAR subunits from the early endosome, there was a significant decrease of IFNAR1/IFNAR2 association in control cells. Conversely, in VPS35-depleted cells, we measured an increased level of association between the two subunits, most probably as a result from a delay in the separation of the IFNAR subunits at the early endosome. Indeed, confocal immunofluorescent microscopy analysis of IFNAR1 and IFNAR2 localization after IFNAR endocytosis revealed a time-dependent increase of the amount of IFNAR2 subunits present in early endosomes after IFN stimulation in VPS35-depleted cells in contrast to control cells where it decreased (Fig. 4b–d).

**Figure 5:**
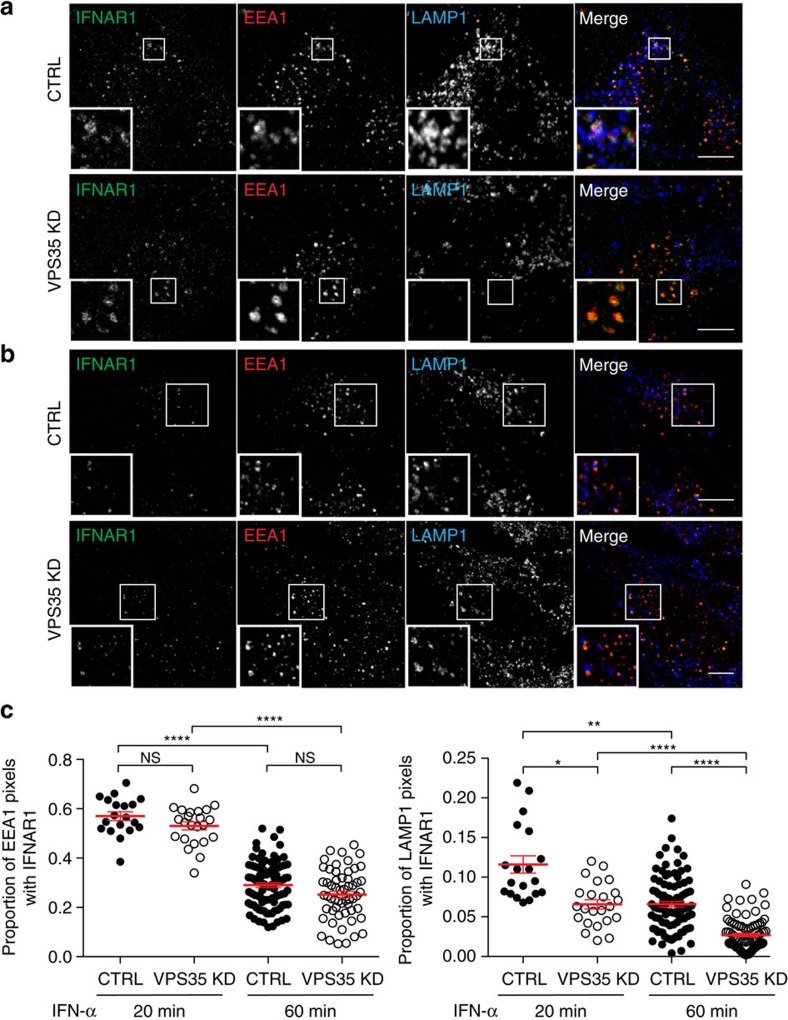
IFNAR1 intracellular trafficking in VPS35-depleted RPE1 cells. (**a**,**b**) Immunofluorescent labelling of endocytosed IFNAR1 in CTRL and VPS35-depleted cells after 20 min (**a**) and 60 min (**b**) of IFNAR uptake under IFN-α stimulation. Cells were fixed, co-labelled for EEA1 and LAMP1, and analysed by confocal microscopy. (**c**) Quantification of co-localization in **a**,**b** expressed as the Manders' coefficient, indicating the proportion of EEA1 pixels (left) or LAMP1 pixels (right) containing IFNAR1 pixels. Scale bars, 10 μm. Reproducibility of experiments: (**a**,**b** and **c**) (20 min: *n*=19, *n*=23 cells respectively; 60 min: *n*=95, *n*=56 cells, respectively) show representative data for three independent experiments. Mean value±s.e.m. Graph **c** left panel: statistical analysis with one-way analsysis of variance and Sidak's multiple comparison test was performed; right panel: statistical analysis with Kruskal–Wallis test was performed. *P<0.05, **P<0.01 and ****P<0.0001; NS, nonsignificant.

In agreement with the lack of direct interaction between IFNAR1 and VPS35, there was no modification of the amount of endocytosed IFNAR1 subunits present in early endosomes after VPS35 depletion at early and late times of endocytosis (Fig. 5a–c). Interestingly, VPS35 depletion also decreased the amount of IFNAR1 subunits sorted to the LAMP1-positive degradative compartments (Fig. 5b,c). These data therefore support a role for VPS35 in the timely control of endosomal sorting of the endocytosed IFNAR subunits, for example, IFNAR2 recycling to the plasma membrane and IFNAR1 degradation in the lysosome. These results are also in agreement with the increased residency of IFNAR2 in EEA1-positive endosomes, in cells depleted from the Rab11A and Rab4A GTPases (Fig. 2). The Rab7 GTPase has been reported to interact with the VPS35/29/26 complex and to mediate the recruitment of the retromer complex to the endosome^21^. We therefore analysed the distribution of IFNAR2 in cells depleted from Rab7A and found that after 20 and 60 min of IFNAR endocytosis in the presence of IFN-α/β, IFNAR2 was significantly accumulated in enlarged endosomes positive for EEA1 (Supplementary Fig. 3a–c). A similar abnormal accumulation of total IFNAR2 was observed at steady state in Rab7A-depleted cells (Supplementary Fig. 3d). These data further establish the role of the retromer complex in IFNAR endosomal sorting and are consistent with the role of Rab7 in retromer recruitment. In support of these results, we previously reported that IFNAR1 was abnormally accumulated in enlarged early endosomes in Rab7A-depleted cells^22^. Altogether, our results indicate that IFNAR1 and IFNAR2 physically segregate from each other at the early endosome, and that VPS35 plays a critical role in this process.

**Figure 6:**
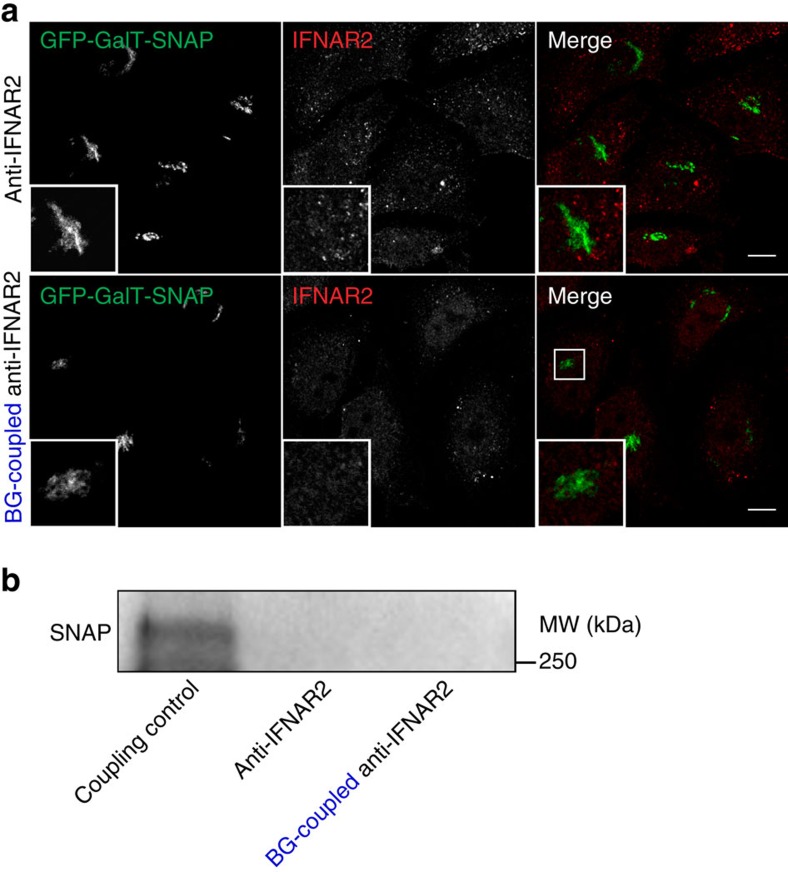
IFNAR2 retrograde trafficking analysis with the SnapTag assay. (**a**) Immunofluorescent labelling of endocytosed IFNAR2 and GFP-GalT-SNAP after 60 min of IFNAR uptake under IFN-β stimulation in HeLa cells stably expressing the GFP-GalT-SNAP construct using either a non-modified anti-IFNAR2 antibody (upper panel) or a BG-coupled anti-IFNAR2 antibody (lower panel). (**b**) Experiments were performed as in **a** and IFNAR2 interaction with the SNAP-tag was revealed by immunoblot analysis. Cell lysates were immunoprecipitated for IFNAR2 and SNAP immunoblotted with anti-SNAP antibody to reveal GalT-GFP-SNAP conjugates. First lane: coupling control: BG-tagged anti-IFNAR2 was incubated with HeLa GFP-GalT-SNAP cell lysate. Second lane: non-modified anti-IFNAR2 antibody. Third lane: BG-coupled anti-IFNAR2 antibody. Scale bars, 10 μm. Reproducibility of experiments: **a** and **b** show representative data for two independent experiments.

The retromer complex has been mainly involved in retrograde trafficking, that is from early endosomes to the *trans*-Golgi network (TGN), for a variety of cargoes including mannose-6-phosphate receptor (MPR), Sortilin-related receptor (SorLA) or Shiga toxin B (refs 23, 24, 25). Although we found that the majority of IFNAR2 was recycling from early endosomes to the plasma membrane (Fig. 2), we could not exclude the possibility that a minor fraction of IFNAR2, not detectable by conventional cell imaging, could transit to the TGN through retromer-dependent sorting. We tested this possibility using the SnapTag approach^26^. The SnapTag fused to the green fluorescent protein (GFP)-tagged galactosyltransferase specifically localized in the TGN (GalT) enables to capture and retain any benzylguanine (BG)-coupled protein that transits, even transiently, through the TGN. We therefore performed IFN-mediated uptake of BG-tagged anti-IFNAR2 antibodies for 60 min at 37 °C followed by subcellular localization analysis by immunofluorescence (IF) and co-immunoprecipitation experiments. Figure 6a shows the absence of co-localization between endocytosed IFNAR2 and GalT-GFP-SNAP, and the co-immunoprecipitation experiments shown in Fig. 6b confirms that IFNAR2 is not present in the TGN, which indicates that IFNAR2 does not follow retrograde trafficking.

### Retromer sorting tunes IFNAR signalling and gene transcription

**Figure 7:**
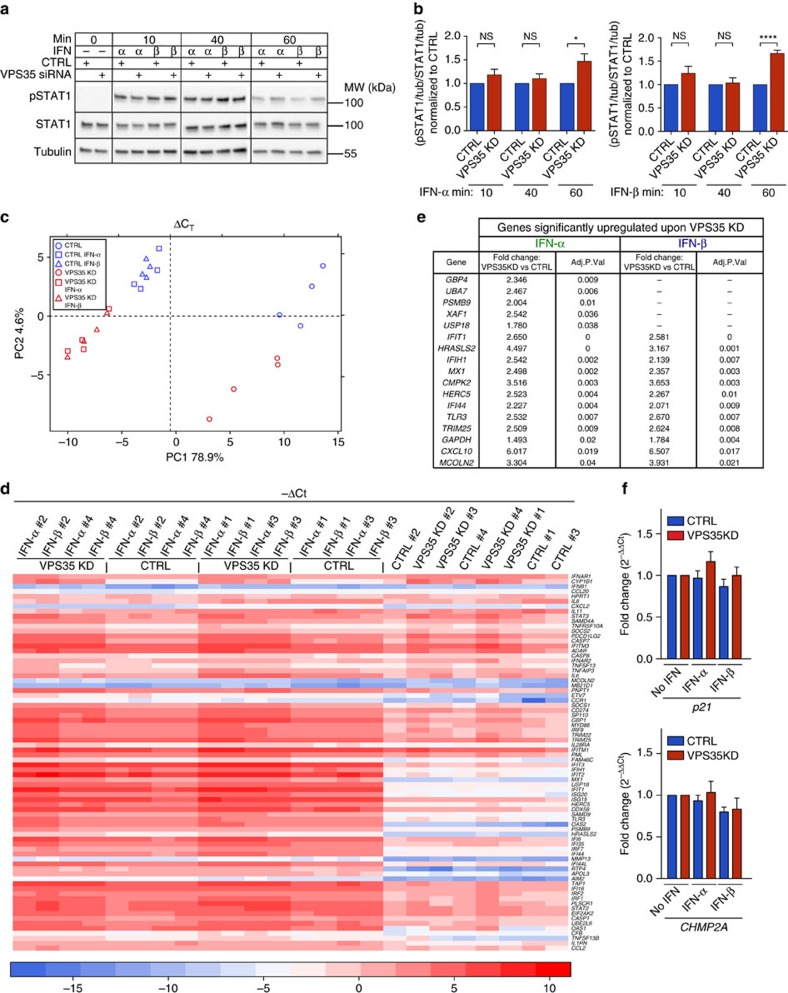
VPS35 depletion results in prolonged activation of JAK/STAT signalling by type-I IFNs. (**a**) Immunoblots for tyrosine phosphorylation levels of STAT1 (pSTAT1) in CTRL and VPS35-depleted RPE1 cells stimulated with IFN-α or IFN-β for the indicated times. Representative immunoblot out of four independent experiments. (**b**) Quantification of experiments performed in **a**: pSTAT1 and STAT1 levels were normalized to tubulin (tub) level (loading control) and the ratio (pSTAT1/tub)/(STAT1/tub) was calculated for each condition. STAT1 activation on VPS35 depletion was normalized to CTRL as 1. (**c**) PCA using ΔCT value in CTRL and VPS35-depleted RPE1 cells with or without IFN-α or IFN-β stimulation for all replicates (biological duplicates with technical duplicates each). (**d**) Clustering analysis based on −ΔCt values for gene expression in CTRL and VPS35-depleted RPE1 cells with or without IFN-α or IFN-β stimulation for all samples replicates (biological duplicates with technical duplicates each). Each square represents a value for a given gene (row) for a specific condition (column). Genes depicted in blue are expressed at low level (low −ΔCT value); genes depicted in red are expressed at high level (high −ΔCT value). (**e**) Genes significantly and selectively upregulated by IFN-α or IFN-β stimulation in VPS35-depleted RPE1 cells. (**f**) Expression of IFN-independent genes: *p21* (upper panel), and *CHMP2A* (lower panel) in CTRL and in VPS35-depleted RPE1 cells with or without IFN-α or IFN-β stimulation. Reproducibility of experiments: panel **a** shows representative data for four independent experiments, **b** shows quantification of data for four, four and three independent experiments for each time point, respectively. Statistical analysis with two-tailed, unpaired *t*-test. *P<0.05 and ****P<0.0001; NS, nonsignificant. (**c**–**e**) Representative data for two independent experiments. (**f**) Quantification of data for three independent experiments. Graphs **b** and **f** show mean value±s.e.m.

The particular aspect of signalling control through endosomal receptor sorting has not yet been investigated for the JAK/STAT signalling pathway. We recently established that clathrin-dependent IFNAR endocytosis was required for the activation of JAK1 and TYK2 kinases associated to the IFNAR complex, implying that endosomal sorting could play a role in the control of JAK/STAT signalling^11^. Considering the key role that the retromer plays in IFNAR sorting at the early endosome, we next investigated whether VPS35-dependent IFNAR sorting may also control the activation of JAK/STAT signalling by IFN-α/β. In agreement with published data, IFN-α/β stimulation of RPE1 cells led to full STAT1 activation (that is, tyrosine phosphorylation) with a maximum observed after 40 min and a decrease of stimulation thereafter (Fig. 7a). In VPS35-depleted cells however, we found an overall increase of STAT1 activation for both IFN-α and IFN-β stimulation (Fig. 7a,b). The increase of STAT1 tyrosine phosphorylation was particularly significant after 60 min of stimulation with IFN-α/β, a time where tyrosine-phosphorylated STAT1 is usually barely detectable in control cells. These results indicate that reducing IFNAR2 interaction with the retromer at the early endosome results in abnormally prolonged activation of STAT1. It is therefore likely to be that the persistent activation of STAT1 results from the aberrantly prolonged association between the two IFNAR1 and IFNAR2 subunits at the early endosome that we observed under VPS35 depletion. We could confirm that increased residency of the IFNAR complex at the early endosome results in prolonged STAT1 activation, as similar results were found in Rab7A-depleted cells where endosomal residency of IFNAR1 and IFNAR2 was also abnormally prolonged (Supplementary Figs 3a–d and 4a,b, and ref. 22). In line with pSTAT1 prolonged activation, VPS35 depletion resulted in prolonged activation of the tyrosine kinase TYK2, which acts upstream of STAT1 (Supplementary Fig. 4c).

Under normal conditions, the tyrosine phosphorylation of cytoplasmic STAT1 and STAT2 results in their dimerization and their translocation to the nucleus where they further associate with IFN-regulatory factor 9 to form the ISG factor 3 complex that binds IFN-sensitive responsive elements in the DNA and initiate gene transcription^1^. We therefore analysed whether the prolonged activation of STAT1 may affect the gene expression profiles induced by IFN stimulation. Gene expression profiles were assessed with the BioMark real-time PCR system that allows to perform high-throughput analysis of 96 complementary DNA samples with 96 different primer pairs in a single run^4^. Primers were chosen for ISGs that represent a broad set of genes involved in the antiviral, antiproliferative and immuno-modulatory activities of type-I IFNs. In addition to RPE1 cells, the profile of gene expression was also investigated in WISH cells, a cell line that is classically used in IFN studies, and allows to extend our findings to cells with different IFNAR numbers or IFN-binding affinity. Cells depleted or not of VPS35 were stimulated with or without 10,000 U ml^−1^ IFN-α or 3,000 U ml^−1^ IFN-β for 6 h, to ensure maximal antiviral and antiproliferative responses. In addition, 6 h of IFN stimulation, although sufficient to elicit proper ISGs expression, allowed to avoid possible biases due to the USP18-mediated negative feedback control observed only after 8 h of IFN-α stimulation^27^,^28^. Gene expression profiles were analysed by several bioinformatics methods. Differences in ISGs expression between control and VPS35-depleted RPE1 cells were first analysed by principal component analysis (PCA) and hierarchical gene clustering. Both analyses included all samples for each condition. PCA was performed on ΔCt values, representing absolute levels of the ISGs (normalized to the level of endogenous control *HPRT1* in each sample), and revealed a good degree of separation between unstimulated and IFN-stimulated conditions (Fig. 7c). Likewise, ISGs expression profiles of control and VPS35-depleted cells were also clearly separated in IFN-stimulated cells (Fig. 7c). In line with the PCA data, the analysis of hierarchical gene clustering (performed on −ΔCt values) revealed that unstimulated control and unstimulated VPS35-depleted cells clustered together and were separated from IFN-stimulated cells (Fig. 7d). Importantly, cells depleted from VPS35 were characterized by higher total gene expression level when comparing with cells normally expressing VPS35. This difference is visible on the heatmap as a red colouring for VPS35 depletion versus light red colouring in control cells for the majority of the genes and confirmed VPS35-dependent expression of the ISGs (Fig. 7d). Under these conditions, we found a list of specific ISGs that were significantly upregulated in VPS35-depleted cells only after IFN-α or IFN-β stimulation in RPE1 cells (Fig. 7e). Similar PCA and heatmap were obtained in WISH cells (Supplementary Fig. 5a–c). Importantly, we did not observe differences between control and VPS35-depleted cells for genes that are known to be independent from IFN stimulation—*p21* and *CHMP2A* (Fig. 7f and Supplementary Fig. 5d). Finally, comparison between RPE1 and WISH cells permitted us to identify a common set of genes differentially expressed on VPS35 depletion in both cell types, therefore ruling out cell-specific particularities (Supplementary Fig. 6a–c).

## Discussion

The JAK/STAT signalling pathway was discovered more than 20 years ago, for its central role in the control of gene activation induced by IFNs. Much attention has since been given to this evolutionary conserved signalling pathway that serves an essential function as a signal transducer downstream of several cytokines, growth factors, hormones and other related proteins^29^. Owing to its important and pleiotropic roles in health and disease, the JAK/STAT signalling pathway is now considered as a paradigm for cell signalling and clinical science^30^. Although this pathway is often presented as a simple and straightforward mechanism by which signalling receptors at the plasma membrane can control gene transcription in the nucleus, its regulation remains nevertheless intrinsically complex. This complexity is particularly well illustrated by type-I IFN, the historical cytokine that led to the discovery of the JAK/STAT pathway. Indeed, 20 years later, we still have a limited comprehension of how 17 different type-I IFNs can transduce common and distinct signalling and biological activities through binding to a single IFNAR receptor complex and by activating shared JAK kinases and STAT molecules. Most studies that have attempted to understand this so-called paradox of signalling have focused on IFN receptor transduction at the plasma membrane^1^,^2^. In agreement with this model, it was recently shown that differential IFN activities were controlled by different affinities towards IFNAR subunits, which in turn conditioned complex stability and conformational changes, and thereby differential receptor internalization rates and STAT activation^5^,^31^.

Another possible layer of regulation that has been so far overlooked for the JAK/STAT signalling pathway is through membrane trafficking of the associated signalling receptors. Indeed, the existence of a tight nexus between receptor trafficking and intracellular signalling has been progressively recognized since early studies on epidermal growth factor receptor endocytosis^10^,^32^. More recent examples including other tyrosine kinase receptors and G protein-coupled receptors have contributed to establish the importance of this new paradigm in mammalian cells and other living organisms, and have identified endosomes as signalling platforms that can be actively engaged in this process^33^,^34^. We previously published that receptor-mediated endocytosis can selectively control the activation of STAT1 within IFNs. Whereas both IFN-γ and IFN-α/β receptors internalization was mediated by clathrin-dependent endocytosis, the selective inhibition of receptor uptake led to a significant decrease of STAT1 activation by IFN-α/β but not IFN-γ^11^. These results indicated that JAK/STAT activation by IFN-γ occurred at the plasma membrane, whereas activation by IFN-α/β was connected to the trafficking of the activated IFNAR complex within the endosomal network^9^. Thus, in addition to the IFNAR structural changes initiated at the plasma membrane, IFNAR endocytosis and trafficking can also play a key role in the control of JAK/STAT signalling induced by IFNs. If there is now consensus that endosomes can actively control the signalling outputs of a growing number of receptors, the underlining molecular machineries remain poorly characterized^7^,^8^. Owing to the intrinsic complexity of intracellular signalling and membrane trafficking, it is likely to be that multiple endosomal sorting machineries are involved^10^.

**Figure 8:**
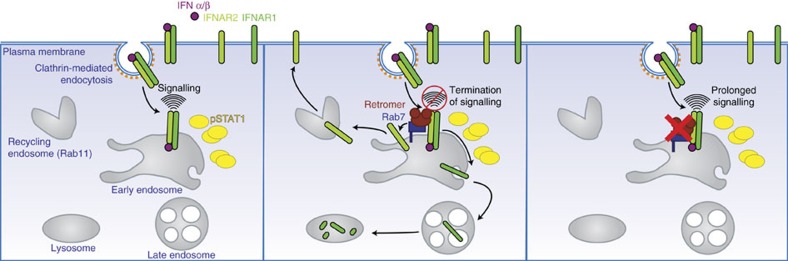
Model for the spatiotemporal control of FNAR endosomal sorting and JAK/STAT signalling by the retromer. Left: at the plasma membrane, the IFNAR complex, assembled by type-I IFNs binding to IFNAR2 and to IFNAR1, is internalized by clathrin-dependent endocytosis, which triggers activation of the JAK kinases and STAT1 phosphorylation (pSTAT1). Middle: the VPS26-29-35 cargo recognition retromer complex is recruited via Rab7 to the early endosome and controls the timely separation of IFNAR1–IFNAR2 subunits that is required for JAK/STAT signalling termination. Right: inhibiting retromer activity results in prolonged IFNAR1-IFNAR2 association at the early endosome, perturbed IFNAR trafficking and abnormally prolonged activation of the JAK/STAT pathway and gene transcription.

In this study we identified the retromer complex as a new factor in JAK/STAT signalling that controls both IFNAR sorting and temporal activation at the early endosome (Fig. 8). The retromer complex is a highly conserved complex that operates by selective recognition of cargo proteins delivered by endocytosis to endosomes^16^,^17^. There is a growing list of cargos that are being actively sorted at the endosomal network through the retromer complex. The majority of these cargos however are recruited by the retromer for their efficient transport between endosomes and the TGN. Using the SNAP-tag approach, we could confirm that IFNAR2 did not undergo retrograde transport from endosomes to the TGN. Instead, IFNAR2 was recycled to the plasma membrane, most probably through the recycling endosome, as this process was inhibited by silencing Rab11A, a master regulator of recycling to the plasma membrane^35^. Cargo selection occurs through binding to the retromer cargo-selective complex comprising VPS35, VPS29 and VPS26 in a process mediated by Rab7A as shown in this study for IFNAR2. Whether and which intracytoplasmic domains of IFNAR2 are involved in VPS35 binding remains unknown. In the case of MPR and SorLA, short aromatic-containing hydrophobic motifs present in the unstructured cytoplasmic tail of these transmembrane receptors have been shown to mediate the interaction with the retromer but no consensus sequence has been clearly identified^36^,^37^. Reversible palmitoylation of cysteine residues present in the tail of retromer-interacting cargos may also play a role in the recognition as suggested for MPR and SorLA^16^. We previously published that IFNAR1 and IFNAR2 were palmitoylated, and that IFNAR1 palmitoylation was required for JAK/STAT signalling^38^. Whether IFNAR2 palmitoylation is involved in retromer recognition is an interesting possibility yet to be tested. Finally, IFNAR recognition by the retromer may represent a new paradigm due to the structural characteristics of the IFNAR complex itself. Indeed, the current dogma of IFNAR activation conveys that the heterodimerization of the IFNAR1/IFNAR2 subunits induced by IFN-α binding at the plasma membrane leads to the activation of IFNAR-associated JAK kinases^5^. It is therefore interesting to speculate that the IFNAR1/IFNAR2 conformational changes induced by IFN-α/β binding would result in increased interaction of IFNAR2 with the retromer at the endosome, whereas it would be less efficient at steady state in the absence of IFN binding. Our data support this hypothesis, as IFNAR1 does not interact with the retromer subunits at steady state and as the interaction of IFNAR2 with VPS35 and VPS26 increases after IFNAR endocytosis.

Whether and how the retromer complex may contribute to the regulation of intracellular signalling remains poorly explored. The retromer could simply regulate signalling by controlling the number of signalling receptors present at the plasma membrane for subsequent rounds of ligand engagement. This is certainly one aspect of IFNAR regulation, as we show here that the retromer controls IFNAR2 recycling to the plasma membrane and, indirectly, IFNAR1 lysosomal degradation. In addition, we also found that the retromer controls the time of residency of the activated IFNAR complex at the early endosome by regulating the timely dissociation of IFNAR2 from IFNAR1, which in turn allows a precise tuning of JAK/STAT signalling termination.

This is somehow reminiscent of a mechanism recently described for GPCRs that also use the retromer for efficient receptor recycling to the plasma membrane through the association with yet another complex called Wiskott–Aldrich syndrome protein and scar homologue^39^. In this case, it was shown that the parathyroid hormone receptor (PTHR) continues to stimulate cyclic AMP production even after receptor internalization^40^. The prolonged production of cAMP was found to be correlated with the persistence of arrestin-receptor complexes on endosomes until retromer turned off PTHR signalling by competing with arrestin for binding to internalized PTHR at the endosome.

In conclusion, our study identifies the first sorting machinery at the crossroads of IFNAR endosomal trafficking and JAK/STAT signalling. Our data highlight a new mechanism by which the retromer complex is required for the timely separation of the IFNAR1–IFNAR2 subunits at the early endosome, which once perturbed leads to abnormally prolonged activation of the JAK/STAT pathway (Fig. 8). We have therefore established a direct link between retromer-mediated receptor sorting and modulation of intracellular signalling and gene transcription. Impaired retromer functions have been linked to neurodegenerative diseases such as Alzheimer's and Parkinson's diseases. Recently published results showed increased expression of IFN-α and IFN-β in the pre-frontal cortex from Alzheimer patients and IFN type-I response to soluble amyloid was shown to depend on MyD88 and IFN-regulated factor 7 signalling^41^,^42^. Whether amyloid β-induced neurotoxicity and development of Alzheimer's disease could be linked to IFN-mediated signalling upregulation as a consequence of retromer dysfunction, as shown in this study for JAK/STAT signalling, is an interesting possibility that should be tested.

## Methods

### Antibodies and reagents

Mouse anti-clathrin heavy chain (BD Transduction Laboratories, 610500, 1:5,000 for WB); goat anti-EEA1 (Santa Cruz Biotechnology, sc-6415, 1:100 for IF); rabbit anti-EEA1 (Cell Signaling, 2411, 1:100 for IF) mouse anti-IFNAR1 AA3 (Biogen, kind gift of Darren Baker, 1:100 for IF, described previously^38^); mouse anti-IFNAR1 EA12 (Biogen, kind gift of Darren Baker, 1:1,000 for IP); rabbit anti-IFNAR1 (Abcam, ab124764, 1:1,000 for WB and 1:100 for IF); mouse anti-IFNAR2 8F11 (kind gift of Pierre Eid, 1:200 for IF, 1:250 for IP, described previously^38^); mouse anti-IFNAR2 10E10 (kind gift of Pierre Eid, 1:2,000 for WB, described previously^38^); rabbit anti-LAMP1 (Abcam, ab 24170, 1:200 for IF); mouse anti-Rab4 (BD Transduction Laboratories, 610888, 1:1,000 for WB); rabbit anti-Rab7 (Santa Cruz Biotechnology, sc-10767, 1:200 for WB); mouse anti-Rab11 (BD Transduction Laboratories, 610656, 1:1,000 for WB); mouse anti-phospho-STAT1 Tyr 701 (BD Transduction Laboratories, 612132, 1:3,000 for WB); rabbit anti-STAT1 (Cell Signaling, 9172, 1:1,000 for WB); rabbit anti-pTYK2 (Cell Signalling, 9321, 1:1,000 for WB); rabbit anti-TYK2 (Millipore, 06-638, 1:1,000 for WB); rabbit anti-VPS26 (Abcam, ab23892, 1:500 for IF); goat anti-VPS35 (Abcam, ab10099, 1:200 for IF); rabbit anti-VPS35 (kind gift of Juan S. Bonifacino, 1:4,000 for WB); mouse anti-alpha-tubulin (Sigma, clone B512, T5168, 1:5,000 for WB). Secondary antibodies conjugated to Alexa Fluor 488, Cy3, Cy5 or horseradish peroxidase (Beckman Coulter or Invitrogen). Cycloheximide (C4859) and fibronectin (F1141) were purchased from Sigma; Tf–Alexa Fluor 647 conjugate was purchased from ThermoFisher Scientific (T-23366); chloroquine was purchased from Sigma (C4859).

### Cell culture

All cells were grown at 37 °C under 5% CO_2_ and routinely tested for the mycoplasma contamination. HeLa cells stably expressing GalT-GFP-SNAP^26^ were cultured in DMEM high-glucose Glutamax (Gibco, Life Technologies), supplemented with 10% FCS (v/v) (Gibco, Life Technologies), 5 mM pyruvate (v/v) (Gibco, Life Technologies) and 1% penicillin–streptomycin (v/v) (Gibco, Life Technologies). hTERT-RPE1 (human retinal pigmented epithelial) cells (kind gift of P. Benaroch) were cultured in DMEM/F12 Glutamax (Gibco, Life Technologies), supplemented with 10% FCS. WISH cells (kind gift of D. Novick) were grown in MEM GlutaMAX (Gibco, Life Technologies) supplemented with 10% FCS, 5 mM pyruvate and 1% penicillin–streptomycin. WISH cells, which remain one of the most used cells in the IFN field, allowed to confirm and extend the role of the retromer on JAK/STAT signalling in another cell type. All cell lines were routinely tested for mycoplasma contamination.

### IFN mutants expression and purification

IFNα2-α8tail-R120E and IFNα2-YNS-M148A were expressed in *Escherichia coli* and purified as described^28^. IFN mutants were site-specifically labelled via the ybbR-tag using CoA conjugated to DY647 (CoA647) catalysed by the PPTase Sfp and further purified by size exclusion chromatography as described^43^. A degree of labelling >90% was obtained for all IFNα2 proteins as determined by ultraviolet–visible spectroscopy.

### RNA interference-mediated silencing

hTERT-RPE1 cells were transfected with small interfering RNAs (siRNAs) using HiPerFect (Qiagen) according to the manufacturer's instructions and cultured for 48 or 72 h as indicated. WISH cells were transfected with siRNAs using INTERFERin (Polyplus transfection) and cultured for 72 h. Experiments were performed on validation of silencing efficiency by immunoblot analysis using specific antibodies and normalizing to the total level of tubulin or clathrin heavy chain used as loading controls. All siRNAs were purchased from Qiagen, apart from Rab7 siRNA (Dharmacon). Control (CTRL) siRNA (SI03650325 5′-AATTCTCCGAACGTGTCACGT-3′) served as reference point and for each experiment was used at the same concentration than siRNAs for the protein of interest. Rab4A siRNA was a kind gift of Graça Raposo (5′-AAGCCAGAACATTGTGATCAT-3′) and used at 10 nM concentration. Two Rab11A siRNAs (SI02663206, 5′-AAGAGCGATATCGAGCTATAA-3′ and SI04437881, 5′-AAGGCTGTGTATAGTCCATTT -3′) were used simultaneously at 10 nM concentration each. For IFNAR1 depletion, three siRNAs sequences were used (SI00013174, 5′-CTGGAATTTGCAATCACTGAA-3′; SI00013195, 5′-TGCCAGAAAGATGATCCCTAA -3′; and SI03065104; 5′-CAGATTGGTCCTCCAGAAGTA-3′), at 5 nM total concentration. To deplete VPS35, GeneSolution pool was used (SI04268181, 5′-CACCATACTCCTTTCCATGTA-3′; SI04279296, 5′-AGCACTTATCTTGGCTACTAA-3′; SI04287605, 5′-CAGAATTGCCCTTAAGACTTT-3′; and SI04316914, 5′-TTGCTGCATCCAAACTTCTAA-3′) at 10 nM total concentration. ON-TARGET SMART pool (Dharmacon) composed of four different siRNAs was used to deplete Rab7.

### IFN stimulation

Human IFN-α2 and IFN-β (kind gift of Pierre Eid) were used at 1,000 U ml^−1^. Only in case of gene expression assays in RPE1 and WISH cells, cells were treated with IFN-α2 at 10,000 U ml^−1^ and IFN-β at 3,000 U ml^−1^.

### High-throughput quantitative PCR analysis

Cells were lysed using RNeasy Plus extraction kit from Qiagen. Reverse-transcription reaction was performed with 1,000 ng of total RNA per reaction using high-capacity cDNA reverse-transcription kit (Applied Biosystems). High-throughput quantitative PCR (qPCR) was performed with BioMark system designed for thermal cycle using microfluidic chips (48 × 48 Dynamic Arrays; Fluidigm Corporation) and real-time data readout. Pre-amplification reactions were done in a GeneAmp PCR System 9700 (Applied Biosystems), using TaqMan Universal Master Mix and TaqMan PreAmp Master Mix (Applied Biosystems).

For pre-amplification of cDNA samples, 50 ng μl^−1^ of cDNA was used per 20 μl reaction and samples were cycled using the recommended programme for 14 cycles. At the end of the cycling programme the reactions were diluted 1:5. Pre-amplified cDNAs prepared in this way was either used immediately or stored at −20 °C until needed. Validation of the pre-amplification reaction with the gene expression assays used in this study was done on the GeneAmp PCR System 9700 following the protocol as described by the manufacturer. Pre-amplified cDNAs were added to the modified 2 × TaqMan Universal Master Mix, to make the final concentration of Master Mix 1.1 × in the samples. Before loading the samples and assay reagents into the inlets, the microfluidic chip was primed in the NanoFlex 4-IFC Controller. Samples prepared as described were then loaded into each sample inlet of the dynamic array chip and 10 × gene expression assay mix was loaded into each detector inlet. The chip was then placed on the NanoFlex 4-IFC Controller for loading and mixing. After ∼55 min, the chip was ready for thermal cycling and detection of the reaction products on the BioMark Real-Time PCR System. The cycling programme used consisted of 10 min at 95 °C followed by 40 cycles of 95 °C for 15 s and 1 min at 60 °C. Data were analysed using the BioMark Gene Expression Data Analysis software to obtain Ct values.

### Bioinformatic analysis of gene expression

In this study, 96 genes were tested in 4 replicates (RPE1 cells) or 2 replicates (WISH cells) and under different conditions. The genes were analysed as ΔCt (with subtracted value of the housekeeping gene *HPRT1*). To limit the batch effect between replicates, a correction of the overall median was performed and the expression of 100 genes was compared. PCA and hierarchical gene clustering was performed on the ΔCt and −ΔCt values, respectively, after the correction of the overall median. The differential analysis of gene expression levels on the basis of ΔCt in VPS35 depletion versus CTRL condition was carried out with the Limma package in R software^44^,^45^. *P*-values adjusted by Benjamini and Hochberg <0.05 were considered statistically significant^46^.

### qPCR for *CHMP2A* and *p21* genes expression

cDNAs were prepared as described above for high-throughput qPCR analysis. qPCR was performed using 50 ng cDNA per 20 μl of reaction. TaqMan Gene Expression Assays from Applied Biosystem were used: *GAPDH* (Hs02758991_g1), *CHMP2A* (Hs00205423_m1) and *p21* (Hs00355782_m1). Relative expression levels were calculated using ΔΔCT method with fold changes calculated as 2^–ΔΔCT^. Glyceraldehyde 3-phosphate dehydrogenase served as the endogenous control and CTRL siRNA sample as the calibrator sample.

### IFNAR endocytosis assay

IFNAR uptake assay was performed as described previously^11^. Cells were grown on coverslips and incubated on ice for 40 min with IFNAR1 AA3 or IFNAR2 8F11 antibodies. In Rab4 and Rab11 experiments, cells were co-incubated with IFNAR antibodies and Tf-Alexa647 (10 μg ml^−1^). Following two washes in ice-cold PBS, IFNAR endocytosis was started by incubating cells at 37 °C in cell culture medium with IFN and stopped with ice-cold PBS after the indicated time. For immunolabelling, coverslips were first washed with ice-cold PBS and with ice-cold stripping medium (0.2 M glycine and 0.15 M NaCl pH 3.0) three times for 90 s, to remove IFNAR-bound antibodies present at the plasma membrane in the case of Rab4- and Rab11-depleted cells. Cells were fixed with 4% paraformaldehyde (Sigma-Aldrich) for 10 min on ice and excess paraformaldehyde was quenched with 50 mM NH_4_Cl (Sigma-Aldrich) for 10 min at room temperature. Cells were permeabilized with 0.05% saponin (Sigma-Aldrich) in 0.2% BSA in PBS for 15 min. Immunostaining was performed with indicated antibodies and samples were mounted in Mowiol (Biovalley). Images were acquired using Nikon A1R confocal microscope equipped with a CFI Plan Apo VC × 60, numerical aperture (NA) 1.4, oil-immersion objective. For quantification of confocal images, background was substracted and the total fluorescence intensity was measured in marked regions using ImageJ software. Co-localization between markers was quantified by the Manders' coefficient using ImageJ co-localization plugin JACoP^47^. RGB intensity plot was obtained using ImageJ plugin RGB Profiler.

### Single-molecule imaging experiments

Single-molecule imaging of fluorescent IFN mutants by total internal reflection fluorescence microscopy was used to measure concentration of the cell surface IFNAR receptor in RPE1 cells and to compare with the reference cell line HeLa. Cells were seeded for 24 h on glass coverslips previously coated with poly-L-lysine-graft-(polyethylene glycol) copolymer functionalized with RGD peptide, to prevent unspecific binding of dye-conjugated IFNs^28^,^48^. Single-molecule fluorescence imaging was carried out with an inverted microscope Olympus IX71 equipped with a triple-line TIR-illumination condenser and a back-illuminated electron-multiplying CCD (charge-coupled device) camera (iXon DU897D, 512 × 512 pixel, Andor Technology), using × 150, NA 1.45 objective. For quantitative ligand-binding experiments, ^DY647^IFNs were excited by 642 nm laser diode at 0.65 mW (power output after passage of the objective, ∼22 W cm^−2^). Stacks of 50 frames were recorded at 32 ms per frame. All experiments were carried out at room temperature in medium without phenol red, supplemented with an oxygen scavenger and a redox-active photoprotectant to minimize photobleaching^28^. The localization density of engineered IFN mutants could be taken as a measure for IFNAR1 and IFNAR2 cell surface concentration. For quantification of IFNAR binding sites, 4 nM of the respective ^DY647^IFNα2 mutant was incubated in medium without phenol red for at least 5 min and kept in the bulk solution during the whole experiment, to ensure equilibrium binding. The mutant ^DY647^IFNα2-α8tail-R120E was used to quantify the IFNAR2 cell surface concentration (binds to IFNAR2 with sub-nanomolar affinity, but is unable to interact with IFNAR1; *K*_D_>100,000 nM to IFNAR1; *K*_D_=0.3 nM to IFNAR2)^49^. For quantification of the cell surface IFNAR1, the mutant IFNα2-YNS-M148A was used. For this mutant, ligand binding to the cell surface is stabilized by simultaneous interaction with IFNAR1 and IFNAR2 (ternary complex formation). At saturating concentration, it binds predominantly to IFNAR1/IFNAR2 or to IFNAR1, but not to IFNAR2 subunit alone, thus allowing quantification of IFNAR1 densitiy (*K*_D_=30 nM to IFNAR1; *K*_D_=150 nM to IFNAR2)^50^,^51^.

### Single-molecule localization and image rendering

Single-molecule localization was carried out as described^28^. Briefly, the positions of single emitters were determined with sub-pixel precision in a two-step process, which was developed for high-density single-particle tracking^52^. To localize initial emitter positions and simultaneously control the rate of erroneous detection, a statistical test was applied to each image pixel. These initial positions were refined in a second step by maximum likelihood estimation modelling the microscope point spread function as a two-dimensional Gaussian profile. To achieve high detection efficiencies, the signals of identified emitters were removed from the image followed by additional detection cycles. This iteration is stopped as soon as no further statistically significant signal sources can be found^52^. Single-molecule localization was carried out by using the multiple-target tracing algorithm as described in detail^52^.

### Proteomic analysis of the endogenous IFNAR2

*Samples preparation*. Anti-IFNAR2 monoclonal antibody clone 8F11 was covalently crosslinked to the magnetic beads using disuccinimidyl suberate according to the manufacturer's protocol (Pierce Crosslink Magnetic IP/Co-IP Kit). Following RPE1 cell stimulation (10 min, 37 °C with or without IFN, 1,000 U ml^−1^), cells were treated with proteinase K (Ambion, AM2546, 50 μg per condition, 5 min, 4 °C) to digest the cell surface proteins including remaining IFNAR2. This allowed to restrict the analysis to the intracellular and endocytosed fractions of IFNAR2. Reaction was stopped with quenching buffer (phenylmethylsulfonyl fluoride 2 mM, EGTA 2 mM, protease inhibitor cocktail and PBS). Cells were lysed on ice with lysis buffer (1% Tx100 and protease inhibitor cocktail) and kept for 30 min at 4 °C on a rotating wheel. Cleared lysates (16,000 *g*, 10 min, 4 °C) were incubated for 1 h with magnetic beads, followed by 1 h incubation with magnetic beads coupled to anti-IFNAR2 antibody. Elution was performed using glycine buffer (0.1 M and pH 2) and the eluate was neutralized with 1 M Tris/HCl pH 8.5. Samples were denaturated 10 min at 99 °C in 0.07% v/v 2-mercapto ethanol and 0.1% v/v SDS in PBS and deglycosylated for 3 h at room temperature by Peptide *N*-glycosidase-F with 50 μg ml^−1^ PNGase-F (Sigma, 7367). Deglycosylated eluates in 5 × sample buffer (Thermo Scientific) were loaded on SDS–PAGE gels.

### Mass spectrometry analysis

Excised gel slices (seven bands per sample) were washed and proteins were reduced with 10 mM dithiothreitol before alkylation with 55 mM iodoacetamide. Gel pieces were then washed and shrunk off with 100% acetonitrile. In-gel digestion was performed using trypsin (Sequencing Grade Modified) overnight in 25 mM ammonium bicarbonate at 30 °C. Peptide concentration and separation was achieved using an actively split capillary HPLC system (Ultimate 3000 system) connected to an LTQ Orbitrap XL mass spectrometer (Thermo Scientific). The mass spectrometer was set to acquire a single MS scan followed by up to five data-dependent scans (dynamic exclusion repeat count of 1, repeat duration of 30 s, exclusion duration of 180 s and lock-mass option was enabled). The resulting spectra were analysed using the Mascot Software created with Proteome Discoverer (version: 1.3, Thermo Scientific) and the SwissProt Homo sapiens (Human) Protein Database (20,233 sequences). The resulting Mascot result files were further processed by using myProMS^2^ and the estimated false discovery rate by automatically filtering the Mascot score of all peptide identifications was set to 1% (Qvality).

### Immunoblotting

Unless stated otherwise, cells were lysed in sample buffer (62.5 mM Tris/HCl pH 6.0, 2% v/v SDS, 10% glycerol, 40 mM dithiothreitol and 0.03% w/v phenol red). Lysates were analysed by SDS–PAGE electrophoresis, immunoblotted with the indicated primary antibodies and horseradish peroxidase-conjugated secondary antibodies. Chemiluminescence signal was revealed using SuperSignal West Dura Extended Duration Substrate or with SuperSignal West Femto Substrate (Thermo Scientific Life Technologies). Acquisition and quantification were performed with the ChemiDoc MP Imaging System (Bio-rad). For immunoblot analysis of total IFNAR1 and IFNAR2 levels, cells were pretreated with 100 μg ml^−1^ cycloheximide for 1 h at 37 °C and where indicated with 100 μg ml^−1^ chloroquine for 20 min at 37 °C. Cells were stimulated with IFN-α or IFN-β for the indicated times. For immunoblot analysis of STAT1 tyrosine phosphorylation (pSTAT1), cells were stimulated with IFN for the indicated time. Phosphorylated and total STAT1 were quantified and normalized to tubulin levels in the same lysate. The ratio of normalized pSTAT1 to normalized total STAT1 was calculated. For immunoblot analysis of TYK2 tyrosine phosphorylation (pTYK2), cells were stimulated with IFN-α for the indicated time. Phosphorylated and total TYK2 levels were quantified and the ratio of pTYK2 to total TYK2 was calculated.

Whole blots images are provided in Supplementary Fig. 7.

### Immunoprecipitations

Cells were lysed on ice with lysis buffer (25 mM Tris/HCl pH 7.5, 100 mM NaCl, 1% Tx100 and protease inhibitor cocktail) and kept for 1 h at 4 °C on a rotating wheel. Cleared lysates (16,000 *g*, 10 min, 4 °C) were incubated overnight at 4 °C with anti-IFNAR1 EA12 or anti-IFNAR2 8F11 antibody, followed by incubation with 30 μl of protein A/G magnetic beads (Thermo Scientific) for 1 h at 4 °C. Magnetic beads were washed three times in lysis buffer. Elution was performed using glycine buffer (0.1 M and pH 2) and eluate was neutralized with 1 M Tris/HCl pH 8.5. Sample buffer was added and samples were resolved on a SDS–PAGE system followed by immunoblotting.

### Proximity ligation assay

Cells grown on coverslips were incubated on ice with anti-IFNAR1 or anti-IFNAR2 antibody for 40 min and washed twice with ice-cold PBS. Cells were stimulated with IFN-α at 37 °C for the indicated times. Fixation and permeabilization were performed as described above. Cells were incubated with primary antibodies as indicated and processed for the PLA assay following the manufacturer's protocol (Sigma-Aldrich). Briefly, cells were incubated with secondary antibodies conjugated to oligonucleotide primers (labelled as probe PLUS and probe MINUS) for 1 h at 37 °C. Ligase was added and the two olignucleotides were ligated provided that they were in close proximity (<40 nm). In the last stage—rolling circle amplification—polymerase and fluorescently labelled nucleotides were used to create a fluorescent reaction product reflecting protein–protein interactions. Reaction products were visible as fluorescent dots and imaged using epifluorescence inverted microscope Leica DM 6000B equipped with a HCX PL Apo 633, NA 1.40, oil-immersion objective and an electron-multiplying CCD camera (Photometrics CoolSNAP HQ). Fluorescent dots were quantified using ImageJ and ‘find maxima' option with a noise tolerance parameter settled visually. Total numbers of dots on images were normalized to the total numbers of cells visualized by 4,6-diamidino-2-phenylindole staining.

### Snap-tag assay

The SNAP-tag assay was performed as previously described^26^. Briefly, mouse anti-IFNAR2 antibody 8F11 was BG tagged, followed by its uptake during IFN-β-mediated IFNAR2 endocytosis for 60 min at 37 °C in HeLa cells stably expressing GFP-GalT-SNAP. Cells were either prepared for immunofluorescent labelling as described for IFNAR endocytosis and stained with the secondary anti-mouse Cy3 antibodies, mounted and analysed by confocal microscopy, or lysed, and processed for immunoprecipitation and immunoblot analysis with anti-SNAP antibody.

### Statistical analyses

All analyses were performed using Prism 6.0 software (GraphPad Inc.). Normality of data distribution was tested using Shapiro–Wilk test. Unpaired, two-tailed *t*-test was used for the comparison of the means between two conditions or when sample size was too small to test normality of data distribution. For more than two conditions, if normally distributed, one-way analysis of variance was used with Sidak's multiple comparisons test or Dunnett's multiple comparisons test. When data were not normally distributed, the unparametric two-tailed Mann–Whitney test (comparison of the means between two conditions) or Kruskal–Wallis test (more than two conditions) with Dunn's multiple comparisons test was performed. Significance of mean comparison is marked on the graphs by asterisks. Error bars denote s.e.m.

No statistical methods were used to predetermine sample size. Experiments were not randomized. The investigators were not blinded to allocation during experiments and outcome assessment.

### Data availability

The data that support the findings of this study are available from the authors on reasonable request; see author contributions for specific data sets.

## Additional information

**How to cite this article:** Chmiest, D. *et al*. Spatiotemporal control of interferon-induced JAK/STAT signalling and gene transcription by the retromer complex. *Nat. Commun.* **7,** 13476 doi: 10.1038/ncomms13476 (2016).

**Publisher's note:** Springer Nature remains neutral with regard to jurisdictional claims in published maps and institutional affiliations.

## Supplementary Material

Supplementary Information Supplementary Figures 1-7 and Supplementary Table 1

## References

[b1] Ivashkiv L. B. & Donlin L. T. Regulation of type I interferon responses. Nat. Rev. Immunol. 14, 36–49 (2014). 24362405 10.1038/nri3581 PMC4084561

[b2] Platanias L. C. Mechanisms of type-I- and type-II-interferon-mediated signalling. Nat. Rev. Immunol. 5, 375–386 (2005). 15864272 10.1038/nri1604

[b3] de Weerd N. A. & Nguyen T. The interferons and their receptors--distribution and regulation. Immunol. Cell Biol. 90, 483–491 (2012). 22410872 10.1038/icb.2012.9 PMC7165917

[b4] Levin D. . Multifaceted activities of type I interferon are revealed by a receptor antagonist. Sci. Signal. 7, ra50 (2014). 24866020 10.1126/scisignal.2004998 PMC4311876

[b5] Piehler J., Thomas C., Garcia K. C. & Schreiber G. Structural and dynamic determinants of type I interferon receptor assembly and their functional interpretation. Immunol. Rev. 250, 317–334 (2012). 23046138 10.1111/imr.12001 PMC3986811

[b6] Vieira A. V., Lamaze C. & Schmid S. L. Control of EGF receptor signaling by clathrin-mediated endocytosis. Science 274, 2086–2089 (1996). 8953040 10.1126/science.274.5295.2086

[b7] Di Fiore P. P. & von Zastrow M. Endocytosis, signaling, and beyond. Cold Spring Harb. Perspect. Biol. 6, pii: a016865 (2014). 10.1101/cshperspect.a016865 PMC4107983 25085911

[b8] Irannejad R., Tsvetanova N. G., Lobingier B. T. & von Zastrow M. Effects of endocytosis on receptor-mediated signaling. Curr. Opin. Cell Biol. 35, 137–143 (2015). 26057614 10.1016/j.ceb.2015.05.005 PMC4529812

[b9] Claudinon J., Monier M. N. & Lamaze C. Interfering with interferon receptor sorting and trafficking: impact on signaling. Biochimie 89, 735–743 (2007). 17493737 10.1016/j.biochi.2007.03.014

[b10] Gonnord P., Blouin C. M. & Lamaze C. Membrane trafficking and signaling: two sides of the same coin. Semin. Cell Dev. Biol. 23, 154–164 (2012). 22085846 10.1016/j.semcdb.2011.11.002

[b11] Marchetti M. . Stat-mediated signaling induced by type I and type II interferons (IFNs) is differentially controlled through lipid microdomain association and clathrin-dependent endocytosis of IFN receptors. Mol. Biol. Cell 17, 2896–2909 (2006). 16624862 10.1091/mbc.E06-01-0076 PMC1483027

[b12] Kumar K. G., Krolewski J. J. & Fuchs S. Y. Phosphorylation and specific ubiquitin acceptor sites are required for ubiquitination and degradation of the IFNAR1 subunit of type I interferon receptor. J. Biol. Chem. 279, 46614–46620 (2004). 15337770 10.1074/jbc.M407082200

[b13] Kumar K. G. . Site-specific ubiquitination exposes a linear motif to promote interferon-alpha receptor endocytosis. J. Cell Biol. 179, 935–950 (2007). 18056411 10.1083/jcb.200706034 PMC2099190

[b14] Marijanovic Z., Ragimbeau J., van der Heyden J., Uze G. & Pellegrini S. Comparable potency of IFNalpha2 and IFNbeta on immediate JAK/STAT activation but differential down-regulation of IFNAR2. Biochem. J. 407, 141–151 (2007). 17627610 10.1042/BJ20070605 PMC2267396

[b15] Sonnichsen B., De Renzis S., Nielsen E., Rietdorf J. & Zerial M. Distinct membrane domains on endosomes in the recycling pathway visualized by multicolor imaging of Rab4, Rab5, and Rab11. J. Cell Biol. 149, 901–914 (2000). 10811830 10.1083/jcb.149.4.901 PMC2174575

[b16] Seaman M. N. The retromer complex - endosomal protein recycling and beyond. J. Cell Sci. 125, 4693–4702 (2012). 23148298 10.1242/jcs.103440 PMC3517092

[b17] Gallon M. & Cullen P. J. Retromer and sorting nexins in endosomal sorting. Biochem. Soc. Trans. 43, 33–47 (2015). 25619244 10.1042/BST20140290

[b18] Burd C. & Cullen P. J. Retromer: a master conductor of endosome sorting. Cold Spring Harb. Perspect. Biol. 6, pii: a016774 (2014). 10.1101/cshperspect.a016774 PMC3941235 24492709

[b19] Teasdale R. D. & Collins B. M. Insights into the PX (phox-homology) domain and SNX (sorting nexin) protein families: structures, functions and roles in disease. Biochem. J. 441, 39–59 (2012). 22168438 10.1042/BJ20111226

[b20] Soderberg O. . Direct observation of individual endogenous protein complexes *in situ* by proximity ligation. Nat. Methods 3, 995–1000 (2006). 17072308 10.1038/nmeth947

[b21] Seaman M. N., Harbour M. E., Tattersall D., Read E. & Bright N. Membrane recruitment of the cargo-selective retromer subcomplex is catalysed by the small GTPase Rab7 and inhibited by the Rab-GAP TBC1D5. J. Cell Sci. 122, 2371–2382 (2009). 19531583 10.1242/jcs.048686 PMC2704877

[b22] Girard E. . Rab7 is functionally required for selective cargo sorting at the early endosome. Traffic 15, 309–326 (2014). 24329906 10.1111/tra.12143

[b23] Johannes L. & Romer W. Shiga toxins—from cell biology to biomedical applications. Nat. Rev. Microbiol. 8, 105–116 (2010). 20023663 10.1038/nrmicro2279

[b24] Nielsen M. S. . Sorting by the cytoplasmic domain of the amyloid precursor protein binding receptor SorLA. Mol. Cell Biol. 27, 6842–6851 (2007). 17646382 10.1128/MCB.00815-07 PMC2099242

[b25] Seaman M. N. Cargo-selective endosomal sorting for retrieval to the Golgi requires retromer. J. Cell Biol. 165, 111–122 (2004). 15078902 10.1083/jcb.200312034 PMC2172078

[b26] Johannes L. & Shafaq-Zadah M. SNAP-tagging the retrograde route. Methods Cell Biol. 118, 139–155 (2013). 24295305 10.1016/B978-0-12-417164-0.00009-4

[b27] Francois-Newton V., Livingstone M., Payelle-Brogard B., Uze G. & Pellegrini S. USP18 establishes the transcriptional and anti-proliferative interferon alpha/beta differential. Biochem. J. 446, 509–516 (2012). 22731491 10.1042/BJ20120541

[b28] Wilmes S. . Receptor dimerization dynamics as a regulatory valve for plasticity of type I interferon signaling. J. Cell Biol. 209, 579–593 (2015). 26008745 10.1083/jcb.201412049 PMC4442803

[b29] Stark G. R. & Darnell J. E. Jr The JAK-STAT pathway at twenty. Immunity 36, 503–514 (2012). 22520844 10.1016/j.immuni.2012.03.013 PMC3909993

[b30] O'Shea J. J. . The JAK-STAT pathway: impact on human disease and therapeutic intervention. Annu. Rev. Med. 66, 311–328 (2015). 25587654 10.1146/annurev-med-051113-024537 PMC5634336

[b31] Thomas C. . Structural linkage between ligand discrimination and receptor activation by type I interferons. Cell 146, 621–632 (2011). 21854986 10.1016/j.cell.2011.06.048 PMC3166218

[b32] Sorkin A. & von Zastrow M. Endocytosis and signalling: intertwining molecular networks. Nat. Rev. Mol. Cell Biol. 10, 609–622 (2009). 19696798 10.1038/nrm2748 PMC2895425

[b33] Miaczynska M. & Bar-Sagi D. Signaling endosomes: seeing is believing. Curr. Opin. Cell Biol. 22, 535–540 (2010). 20538448 10.1016/j.ceb.2010.05.007 PMC3020151

[b34] Scita G. & Di Fiore P. P. The endocytic matrix. Nature 463, 464–473 (2010). 20110990 10.1038/nature08910

[b35] Welz T., Wellbourne-Wood J. & Kerkhoff E. Orchestration of cell surface proteins by Rab11. Trends Cell Biol. 24, 407–415 (2014). 24675420 10.1016/j.tcb.2014.02.004

[b36] Fjorback A. W. . Retromer binds the FANSHY sorting motif in SorLA to regulate amyloid precursor protein sorting and processing. J. Neurosci. 32, 1467–1480 (2012). 22279231 10.1523/JNEUROSCI.2272-11.2012 PMC6796259

[b37] Seaman M. N. Identification of a novel conserved sorting motif required for retromer-mediated endosome-to-TGN retrieval. J. Cell Sci. 120, 2378–2389 (2007). 17606993 10.1242/jcs.009654

[b38] Claudinon J. . Palmitoylation of interferon-alpha (IFN-alpha) receptor subunit IFNAR1 is required for the activation of Stat1 and Stat2 by IFN-alpha. J. Biol. Chem. 284, 24328–24340 (2009). 19561067 10.1074/jbc.M109.021915 PMC2782026

[b39] Harbour M. E. . The cargo-selective retromer complex is a recruiting hub for protein complexes that regulate endosomal tubule dynamics. J. Cell Sci. 123, 3703–3717 (2010). 20923837 10.1242/jcs.071472 PMC2964111

[b40] Feinstein T. N. . Retromer terminates the generation of cAMP by internalized PTH receptors. Nat. Chem. Biol. 7, 278–284 (2011). 21445058 10.1038/nchembio.545 PMC3079799

[b41] Taylor J. M. . Type-1 interferon signaling mediates neuro-inflammatory events in models of Alzheimer's disease. Neurobiol. Aging 35, 1012–1023 (2014). 24262201 10.1016/j.neurobiolaging.2013.10.089

[b42] Minter M. R. . Soluble amyloid triggers a myeloid differentiation factor 88 and interferon regulatory factor 7 dependent neuronal type-1 interferon response *in vitro*. J. Neuroinflammation 12, 71 (2015). 25879763 10.1186/s12974-015-0263-2 PMC4407532

[b43] Waichman S. . Functional immobilization and patterning of proteins by an enzymatic transfer reaction. Anal. Chem. 82, 1478–1485 (2010). 20092261 10.1021/ac902608a

[b44] Smyth G. K., Michaud J. & Scott H. S. Use of within-array replicate spots for assessing differential expression in microarray experiments. Bioinformatics 21, 2067–2075 (2005). 15657102 10.1093/bioinformatics/bti270

[b45] Wettenhall J. M. & Smyth G. K. limmaGUI: a graphical user interface for linear modeling of microarray data. Bioinformatics 20, 3705–3706 (2004). 15297296 10.1093/bioinformatics/bth449

[b46] Hochberg Y. & Benjamini Y. More powerful procedures for multiple significance testing. Stat. Med. 9, 811–818 (1990). 2218183 10.1002/sim.4780090710

[b47] Bolte S. & Cordelieres F. P. A guided tour into subcellular colocalization analysis in light microscopy. J. Microsc. 224, 213–232 (2006). 17210054 10.1111/j.1365-2818.2006.01706.x

[b48] VandeVondele S., Voros J. & Hubbell J. A. RGD-grafted poly-L-lysine-graft-(polyethylene glycol) copolymers block non-specific protein adsorption while promoting cell adhesion. Biotechnol. Bioeng. 82, 784–790 (2003). 12701144 10.1002/bit.10625

[b49] Pan M. . Mutation of the IFNAR-1 receptor binding site of human IFN-alpha2 generates type I IFN competitive antagonists. Biochemistry 47, 12018–12027 (2008). 18937499 10.1021/bi801588g

[b50] Kalie E., Jaitin D. A., Abramovich R. & Schreiber G. An interferon alpha2 mutant optimized by phage display for IFNAR1 binding confers specifically enhanced antitumor activities. J. Biol. Chem. 282, 11602–11611 (2007). 17310065 10.1074/jbc.M610115200

[b51] Piehler J., Roisman L. C. & Schreiber G. New structural and functional aspects of the type I interferon-receptor interaction revealed by comprehensive mutational analysis of the binding interface. J. Biol. Chem. 275, 40425–40433 (2000). 10984492 10.1074/jbc.M006854200

[b52] Serge A., Bertaux N., Rigneault H. & Marguet D. Dynamic multiple-target tracing to probe spatiotemporal cartography of cell membranes. Nat. Methods 5, 687–694 (2008). 18604216 10.1038/nmeth.1233

