# Supplementary material for: Spatiotemporal control of interferon-induced JAK/STAT signalling and gene transcription by the retromer complex
Source: Nat Commun. 2016 Dec 5;7:13476. doi: 10.1038/ncomms13476 (PMC5150223; doi:10.1038/ncomms13476)
Supplement: Supplementary Information — Supplementary Figures 1-7 and Supplementary Table 1 [file ncomms13476-s1.pdf]

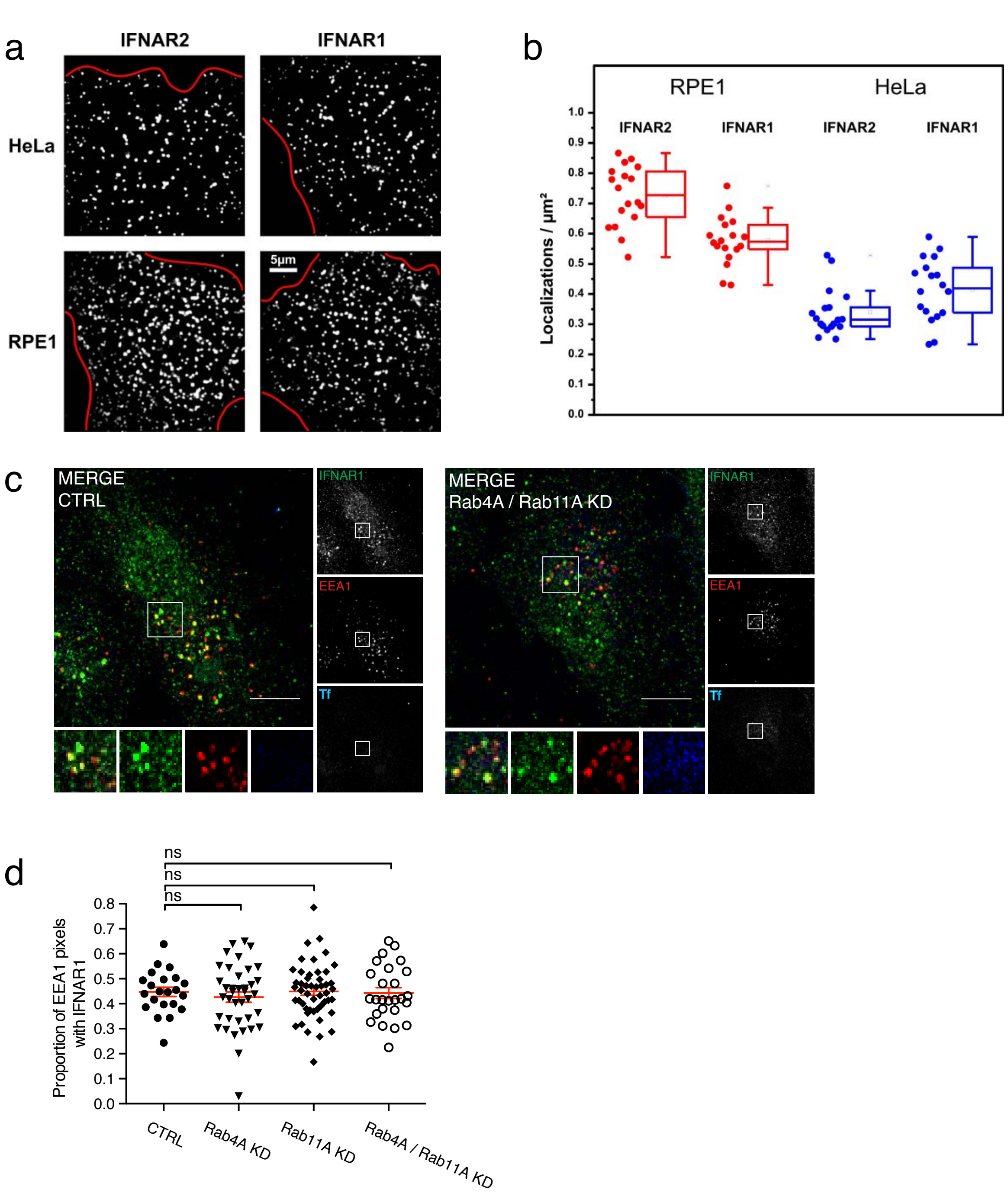

Supplementary Figure 1. (**a-b**) Quantification of IFNAR1 and IFNAR2 subunits cell surface expression in RPE1 and HeLa cells. (**a**) TIRF microscopy images of HeLa and RPE1 cells after addition of 4 nM DY647-conjugated IFN mutants. The localization density of cell-bound ^{DY647}IFNα2-α8tail-R120E was used to determine cell surface IFNAR2 levels whereas ^{DY647}IFNα2-YNS-M148A was taken to quantify IFNAR1 levels. The boundaries of the cells are indicated by a red line. Scale bar 5 μm. (**b**) RPE1 cells express 0,58 IFNAR1 per μm^2 and 0,72 IFNAR2 per μm^2 subunits whereas HeLa cells express 0,41 IFNAR1 per μm^2 and 0,34 IFNAR2 per μm^2 subunits. (**c-d**): IFNAR1 intracellular trafficking is independent from Rab11A and Rab4A. (**c**) Uptake of Transferrin-Alexa647 conjugate and IFNAR1 subunit in control (CTRL) and Rab4A / Rab11A depleted RPE1 cells upon 60 min IFN-α stimulation. Following fixation, cells were co-labeled for EEA1 and analyzed by confocal microscopy. Scale bar 10 μm. (**d**) Quantification of colocalization in **c** expressed as the Manders' coefficient indicating the proportion of EEA1 pixels containing IFNAR1 pixels in CTRL, Rab4A, Rab11A or Rab4A/Rab11A depleted RPE1 cells. **Reproducibility of experiments: a-b** (n=18 cells per condition) show representative data for 2 independent experiments. Data distribution of the second + the third quartile, median (line), mean (empty square) and whiskers (1.5x interquartile range) is shown. **c-d** (n=22, n=38, n=49 and n=26 cells, respectively, for each condition) show representative data for 3 independent experiments. Mean value ± SEM. Statistical analysis with one-way ANOVA and Dunnett's multiple comparison test was performed; ns = non-significant.

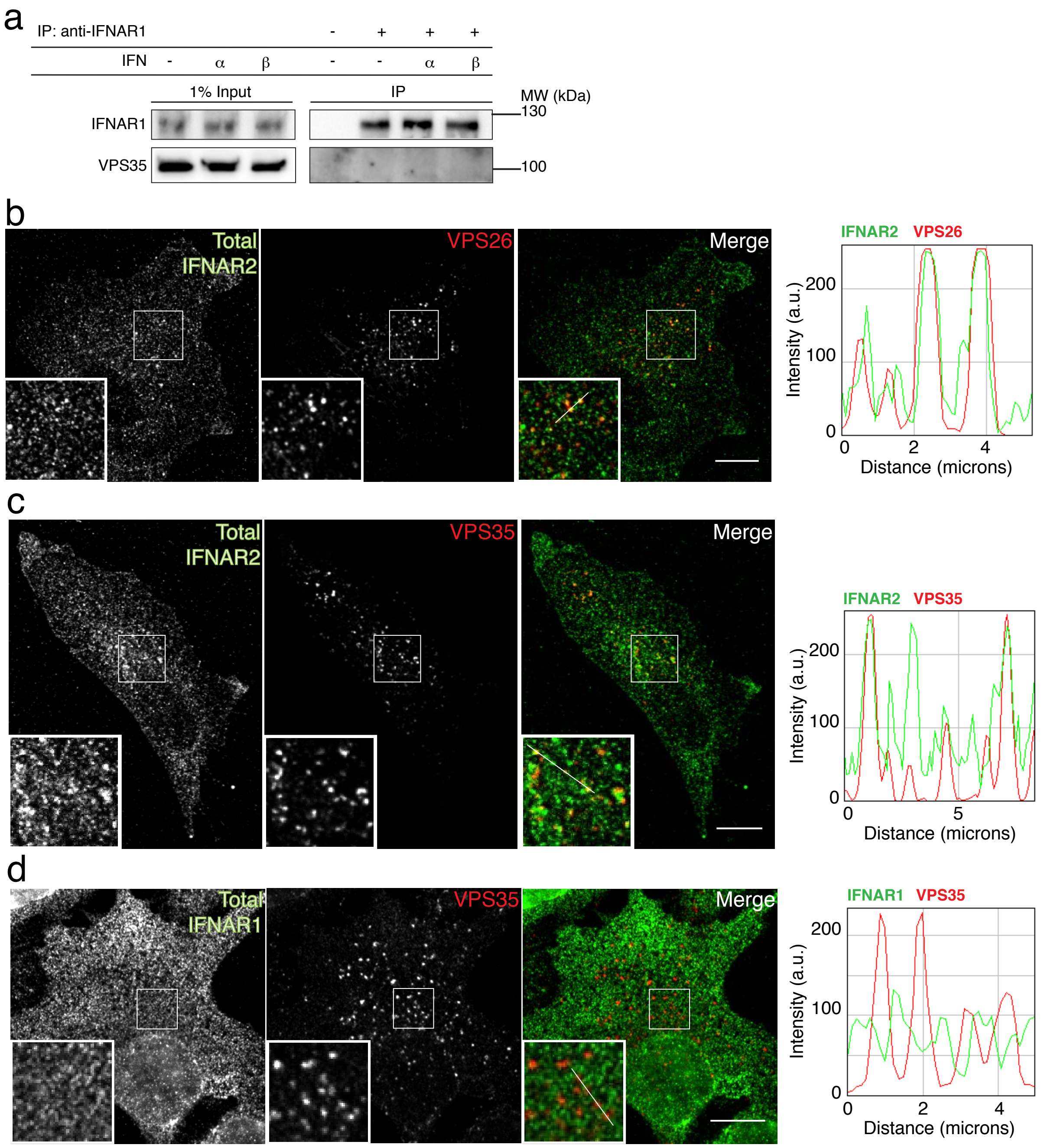

Supplementary Figure 2. IFNAR1 and IFNAR2 interaction with the retromer. (**a**) Untransfected RPE1 cells were stimulated or not (-) with IFN-α or IFN-β as indicated. Cell lysates were incubated with anti-IFNAR1 antibody to immunoprecipitate (IP) endogenous IFNAR1 and reveal co-immunoprecipitated endogenous VPS35. (**b,c**) Immunofluorescent labeling of total IFNAR2 in RPE1 cells with: (**b**) VPS26 with representative histogram of colocalization profile (**c**) VPS35 with representative histogram of colocalization profile. (**d**) Immunofluorescent labeling of total IFNAR1 and VPS35 in RPE1 cells with representative histogram of colocalization profile. Scale bars 10 μm. **Reproducibility of experiments: a-d** show representative data for 3 independent experiments.

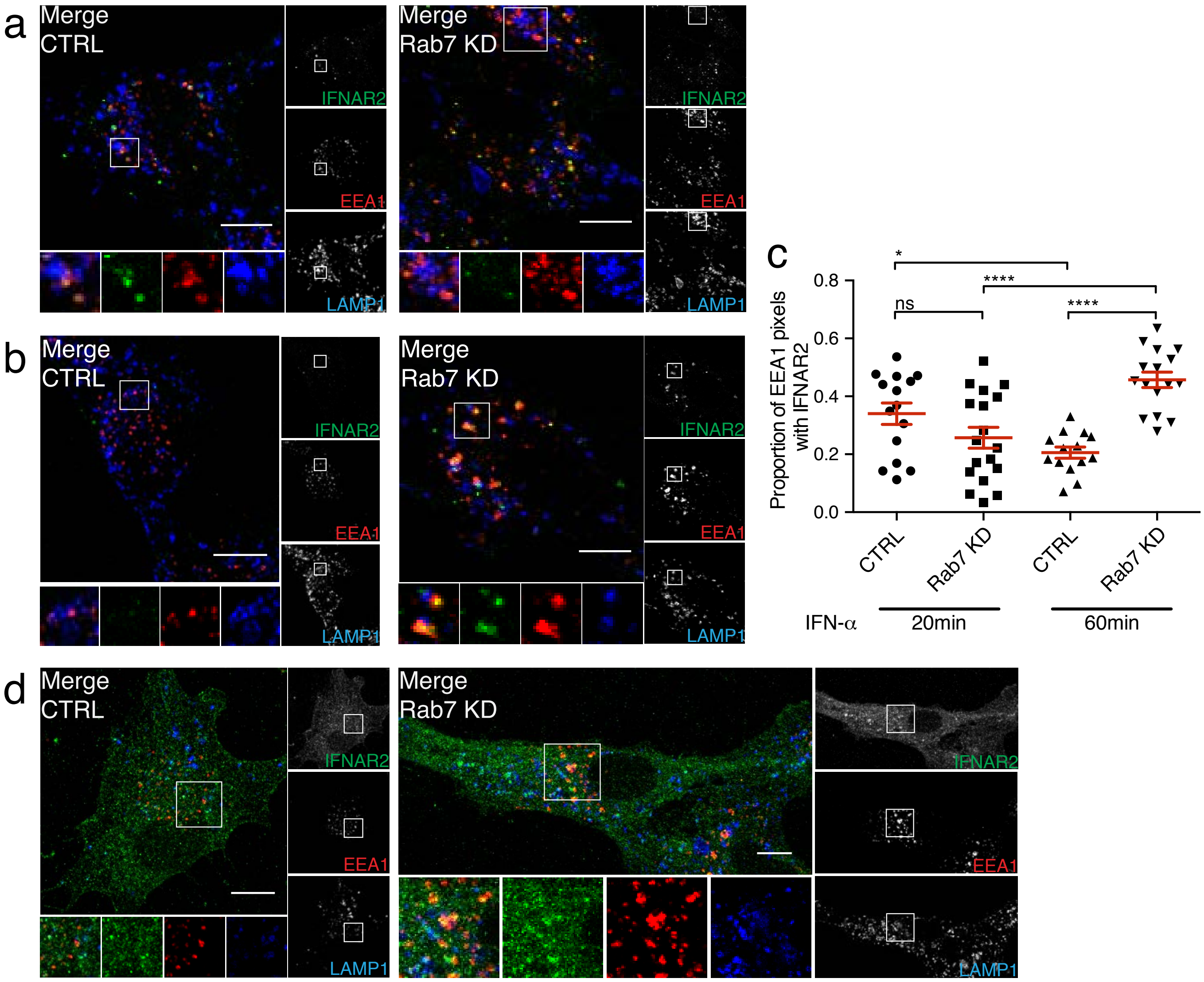

Supplementary Figure 3. Effect of Rab7A depletion on IFNAR2 trafficking in RPE1 cells. (**a**,**b**) Immunofluorescent labeling of endocytosed IFNAR2 in CTRL and Rab7A depleted cells after 20 min (**a**) and 60 min (**b**) IFNAR uptake under IFN-α stimulation. Cells were fixed, co-labeled for EEA1 and LAMP1, and analyzed by confocal microscopy. (**c**) Quantification of colocalization in **a**,**b** expressed as the Manders' coefficient indicating the proportion of EEA1 pixels containing IFNAR2 pixels. (**d**) Immunofluorescent labeling of total IFNAR2, EEA1 and LAMP1 in CTRL and Rab7A depleted cells, observed by confocal microscopy. Scale bars 10 μm. **Reproducibility of experiments: a-d** show representative data for 3 independent experiments. Graph **c** – shows quantification of 20 min: n=15 and n=18 cells respectively; 60 min: n=14 cells and n=16 cells, respectively, for each condition. Mean value ± SEM. Statistical analysis with one-way ANOVA and Sidak's multiple comparison test was performed. **P*<0.05; *****P*<0.0001; ns = non-significant.

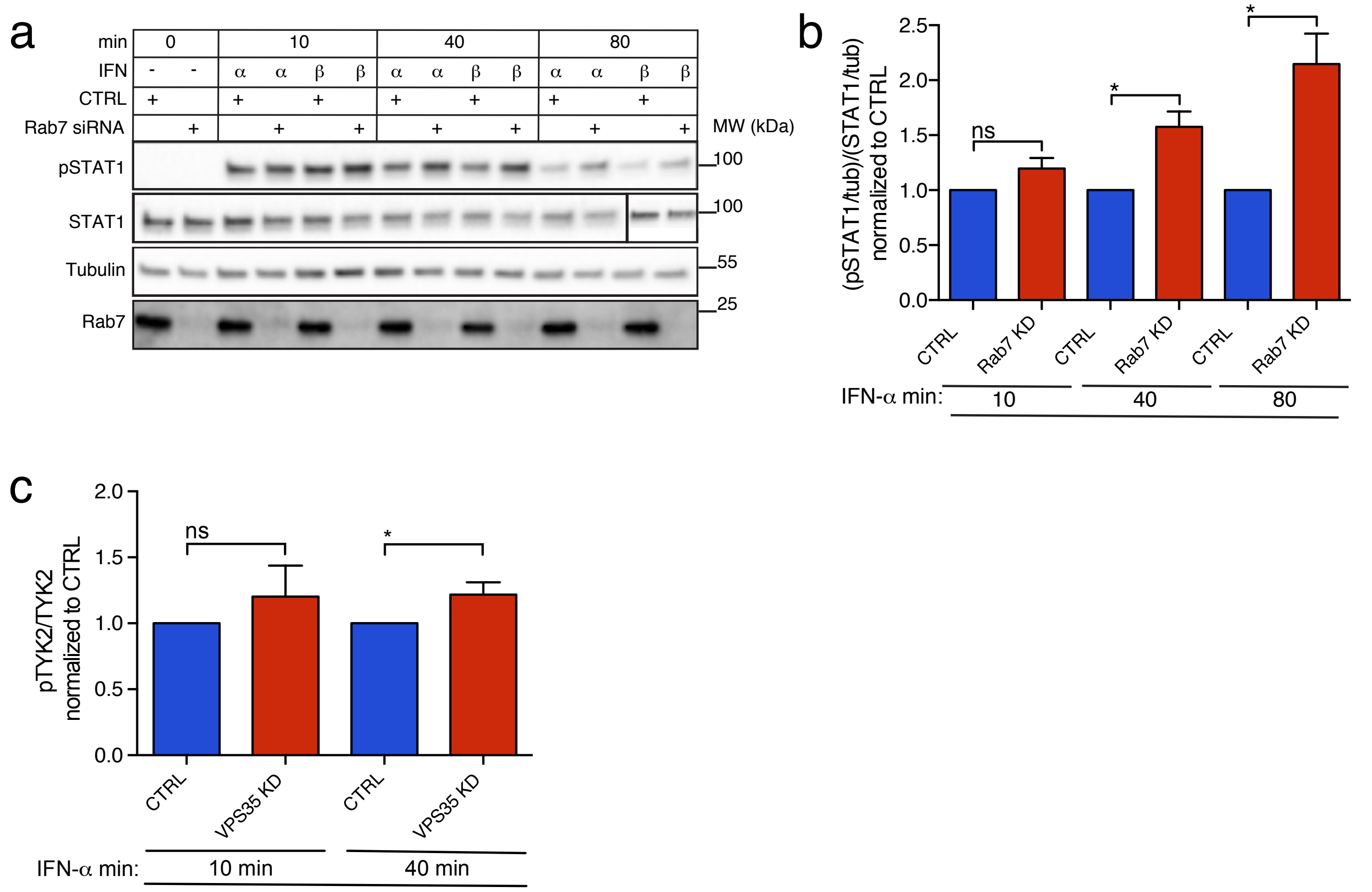

Supplementary Figure 4. Effect of Rab7A and VPS35 depletion on JAK/STAT signaling in RPE1 cells. (**a**) Immunoblots for tyrosine phosphorylation levels of STAT1 (pSTAT1) in CTRL and Rab7A depleted RPE1 cells stimulated with IFN-α or IFN-β for the indicated times. (**b**) Quantification of experiments performed in **a**: pSTAT1 and STAT1 levels were normalized to tubulin (tub) level (loading control) and the ratio (pSTAT1/tub)/(STAT1/tub) was calculated for each condition. STAT1 activation upon Rab7 depletion was normalized to CTRL as 1. (**c**) Quantification of tyrosine phosphorylation levels of TYK2 (pTYK2) in CTRL and VPS35 depleted RPE1 cells stimulated with IFN-α for the indicated times. Ratio of pTYK2 level to the total TYK2 level was calculated for each condition. TYK2 activation upon VPS35 depletion was normalized to CTRL as 1. **Reproducibility of experiments: a** shows representative data for 3 independent experiments, **b** shows quantification of 3 independent experiments and **c** shows quantification of 7 independent experiments. Graphs **b-c** show mean value ± SEM, statistical analysis with two-tailed, unpaired t-test was performed. *P<0.05; ns = non-significant.

a

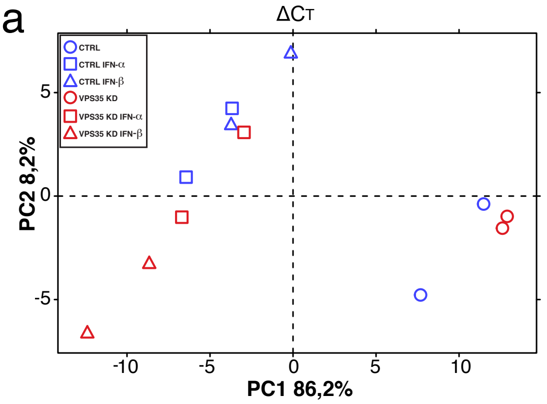

c

Genes significantly upregulated upon IFN-β in VPS35 KD					
Gene	Fold change: VPS35KD vs CTRL	adj.P.Val	Gene	Fold change: VPS35KD vs CTRL	adj.P.Val
ACTB	3,789	0,001	*USP18*	2,309	0,009
HRPS11	3,147	0,001	*SP110*	3,176	0,01
ADAR	2,892	0,001	*TAP1*	3,193	0,01
IFITM1	2,634	0,001	*IL11*	2,476	0,01
PDCD1	4,176	0,001	*IFNAR2*	2,872	0,01
IL6	4,199	0,001	*TRIM25*	3,499	0,01
FAM46C	3,403	0,001	*PDCD1LG2*	2,412	0,013
IFITM3	3,821	0,001	*HRASLS2*	2,360	0,013
EIF2AK2	2,772	0,002	*IFNAR1*	3,593	0,013
ISG15	3,499	0,003	*TNFSF10*	1,730	0,014
OAS1	2,310	0,004	*SOCS1*	2,478	0,015
TLR3	3,352	0,004	*CCR1*	3,193	0,015
PLSCR1	2,403	0,005	*IFIH1*	2,764	0,017
CXCL10	1,867	0,006	*IFNB1*	2,770	0,017
STAT3	3,848	0,006	*SOCS2*	1,679	0,017
IFI44	2,975	0,007	*CASP8*	3,387	0,017
CD274	2,020	0,009	*MB21D1*	1,981	0,017
IL28RA	3,151	0,009	*PSMB9*	2,198	0,017

b

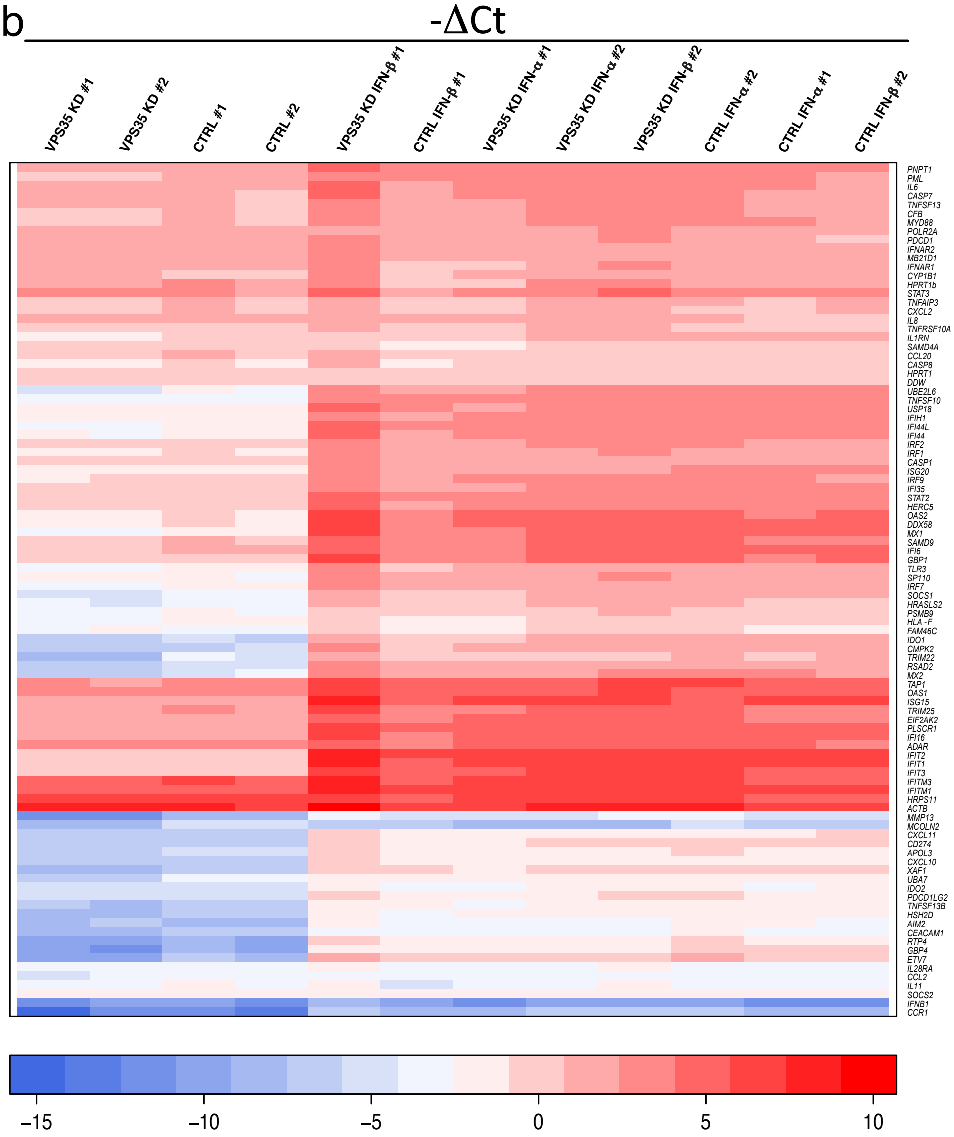

d

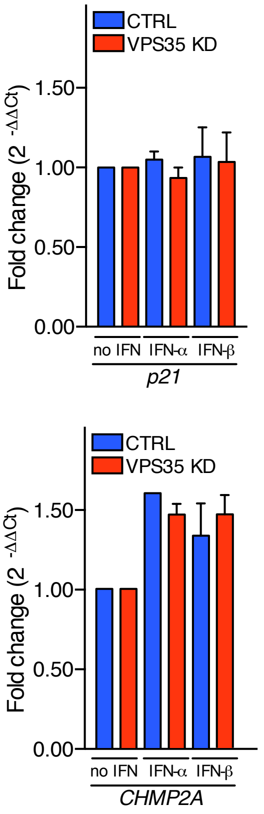

Supplementary Figure 5. Gene expression analysis in WISH cells. (**a**) Principal component analysis (PCA) using ΔCT value in CTRL and VPS35 depleted WISH cells with or without IFN-α or IFN-β stimulation for all samples replicates. (**b**) Clustering analysis based on −ΔCt values for gene expression in CTRL and VPS35 depleted WISH cells with or without IFN-α or IFN-β stimulation for all samples replicates. Each square represents a value for a given gene (row) for a specific condition (column). Genes depicted in blue are expressed at low level (low −ΔCT value); genes depicted in red are expressed at high level (high −ΔCT value). (**c**) Genes significantly upregulated by IFN-β stimulation in VPS35 depleted WISH cells. (**d**) Expression of IFN-independent genes: *p21* (upper panel), and *CHMP2A* (lower panel) in CTRL and in VPS35-depleted WISH cells with or without IFN-α or IFN-β stimulation. Mean value ± SEM. Quantification of 2 independent experiments.

a

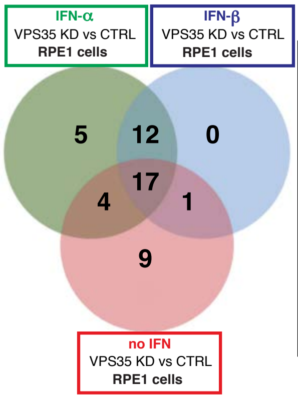

RPE1 cells<br>Genes differentially expressed upon VPS35 KD (vs CTRL)					
IFN-α	no IFN	IFN-α/IFN-β	IFN-α/no IFN	IFN-β/no IFN	IFN-α/IFN-β<br>no IFN
GBP4	RTP4	IFIT1	CFB	ACTB	SAMD9
UBA7	HSH2D	HRASLS2	HLA-F		TNFSF10
PSMB9	AIM2	IFIH1	STAT2		FAM46C
XAF1	CCR1	MX1	TNFAIP3		EIF2AK2
USP18	POLR2A	CMPK2			PDCD1LG2
	IL1RN	HERC5			RSAD2
	PDCD1	IFI44			ISG20
	MMP13	TLR3			IFITM1
	IRF7	TRIM25			CXCL11
		GAPDH			IL28RA
		CXCL10			MB21D1
		MCOLN2			ADAR
					IFNB1
					TNFRSF10A
					CYP1B1
					IFNAR1
					IFITM3

b

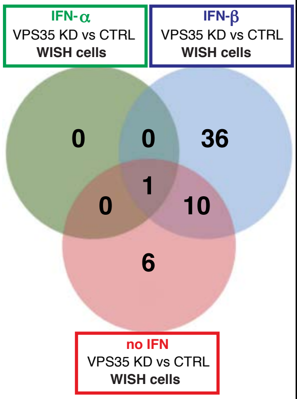

WISH cells<br>Genes differentially expressed upon VPS35 KD (vs CTRL)			
IFN-β	no IFN	IFN-β/<br>no IFN	IFN-α/IFN-β<br>no IFN
ACTB	UBA7	MX2	MCOLN2
HRPS11	TRIM22	CMPK2	
ADAR	ETV7	IFI44L	
IFITM1	CEACAM1	IRF7	
PDCD1	APOL3	XAF1	
IL6	HLA-F	HSH2D	
FAM46C		MX1	
IFITM3		UBE2L6	
EIF2AK2		RSAD2	
ISG15		MMP13	
OAS1			
TLR3			
PLSCR1			
CXCL10			
STAT3			
IFI44			
CD274			
IL28RA			
USP18			
SP110			
TAP1			
IL11			
IFNAR2			
TRIM25			
PDCD1LG2			
HRASLS2			
IFNAR1			
TNFSF10			
SOCS1			
CCR1			
IFIH1			
IFNB1			
SOCS2			
CASP8			
MB21D1			
PSMB9			

c

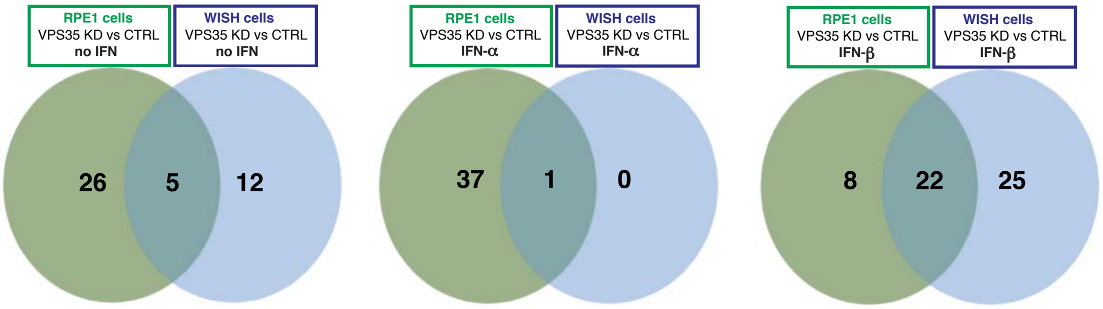

Genes differentially expressed upon VPS35 KD (vs CTRL)							
CTRL vs VPS35 KD no IFN			CTRL vs VPS35 KD IFN-α		CTRL vs VPS35 KD IFN-β		
RPE1	WISH	RPE1 and WISH	RPE1	WISH	RPE1	WISH	RPE1 and WISH
FAM46C	MCOLN2	HSH2D	IFIT1	MCOLN2	IFIT1	HRPS11	FAM46C
TNFSF10	UBA7	RSAD2	SAMD9		SAMD9	MX2	HRASLS2
RTP4	TRIM22	HLA-F	HRASLS2		ISG20	PDCD1	TNFSF10
PDCD1LG2	XAF1	MMP13	TNFSF10		GAPDH	IL6	PDCD1LG2
ADAR	MX2	IRF7	FAM46C		CYP1B1	ISG15	IL28RA
SAMD9	IFI44L		EIF2AK2		TNFRSF10A	OAS1	CMPK2
IFITM3	ETV7		PDCD1LG2		HERC5	IFI44L	ADAR
AIM2	CEACAM1		RSAD2		CXCL11	PLSCR1	MB21D1
MB21D1	UBE2L6		IFIH1			STAT3	IFITM3
CCR1	APOL3		ISG20			IRF7	MX1
ISG20	CMPK2		MX1			XAF1	TLR3
TNFRSF10A	MX1		CMPK2			HSH2D	EIF2AK2
IFNAR1			CFB			CD274	IFIH1
ACTB			IFITM1			USP18	RSAD2
POLR2A			CXCL11			SP110	IFNAR1
IL1RN			IL28RA			TAP1	TRIM25
CXCL11			MB21D1			IL11	IFI44
PDCD1			ADAR			IFNAR2	IFITM1
IFITM1			IFNB1			UBE2L6	CXCL10
IL28RA			TNFRSF10A			MMP13	ACTB
CYP1B1			HERC5			SOCS1	MCOLN2
EIF2AK2			IFI44			CCR1	IFNB1
STAT2			HLA-F			SOCS2	
IFNB1			GBP4			CASP8	
CFB			CYP1B1			PSMB9	
TNFAIP3			UBA7				
			IFNAR1				
			TLR3				
			TRIM25				
			PSMB9				
			GAPDH				
			IFITM3				
			CXCL10				
			STAT2				
			XAF1				
			USP18				
			TNFAIP3				

Supplementary Figure 6. Comparative analysis of IFN-dependent genes in CTRL vs. VPS35 depleted WISH and RPE1 cells. (**a**) RPE1 cells. Venn Diagram representing differentially expressed genes upon stimulation without (red circle) or with IFN-α (green circle), or IFN-β (blue circle) in CTRL vs. VPS35 depleted cells. Table listing the genes that are differentially expressed for each condition in RPE1 cells (right). (**b**) WISH cells. Venn Diagram representing differentially expressed genes without (red circle) or with IFN-α (green circle) or IFN-β (blue circle) stimulation in CTRL vs. VPS35 depleted cells. Table listing the genes that are differentially expressed for each condition in WISH cells (right). (**c**) RPE1 vs. WISH cells. Venn Diagrams representing differentially expressed genes without (left panel) or with IFN-α (middle panel) or IFN-β stimulation (right panel) in CTRL vs. VPS35 depleted cells in RPE1 (green circle) and WISH (blue circle) cells. Table listing the genes that are differentially expressed for each condition (lower panel).

Fig. 1e IFNAR1

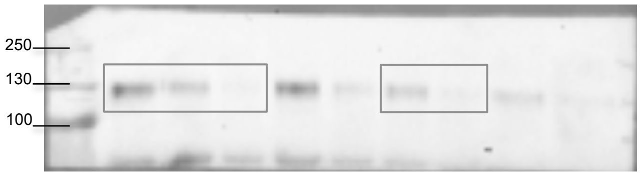

Fig. 1e IFNAR2

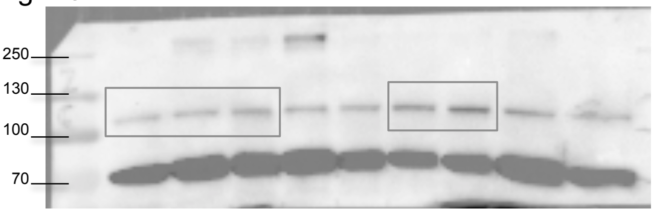

Fig. 1e tubulin

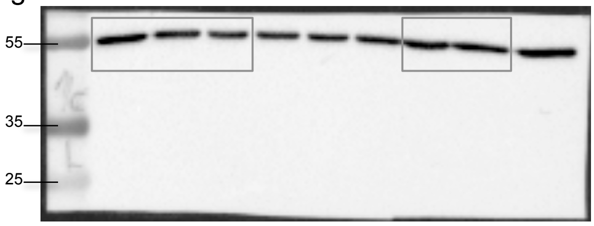

Fig. 1g IFNAR1

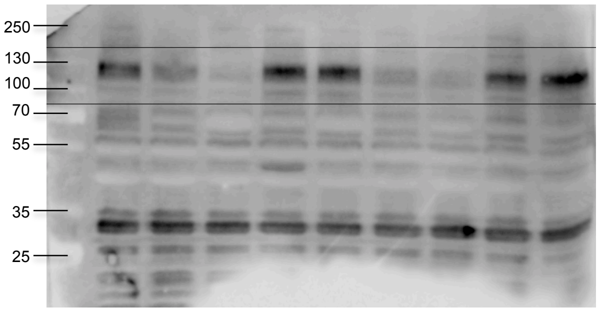

Fig. 2a Rab4

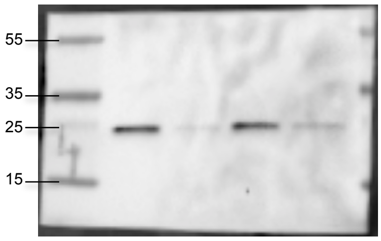

Fig. 2a Rab11

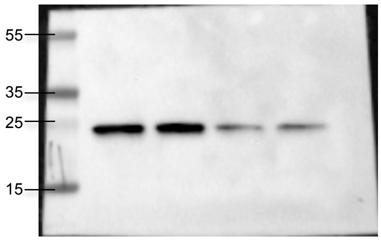

Fig. 2a CHC

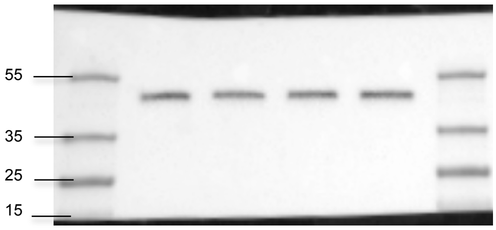

Fig. 3a 1% Input IFNAR2

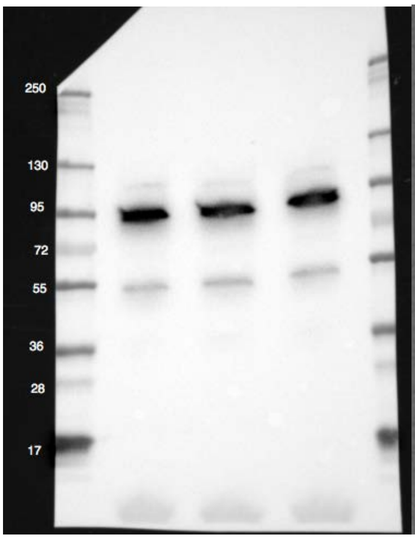

Fig. 3a 1% Input VPS35

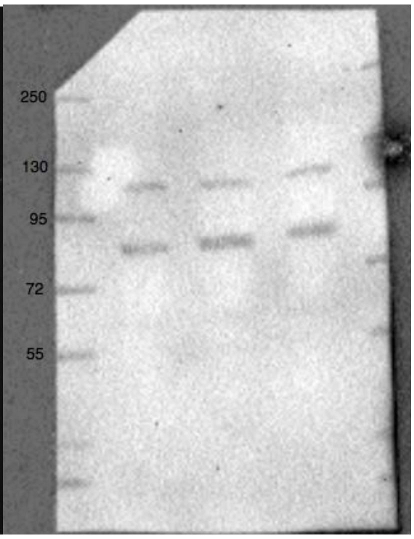

Supplementary Figure 7.

Fig. 3a VPS35 IP

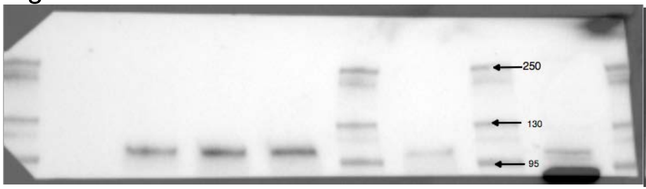

Fig. 3a IFNAR2 IP

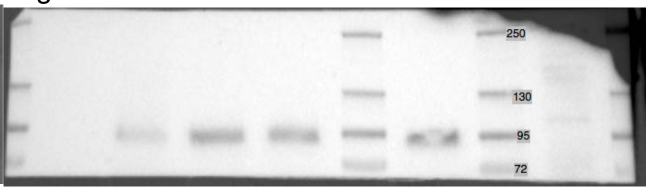

Fig. 3b IFNAR2 1% Input

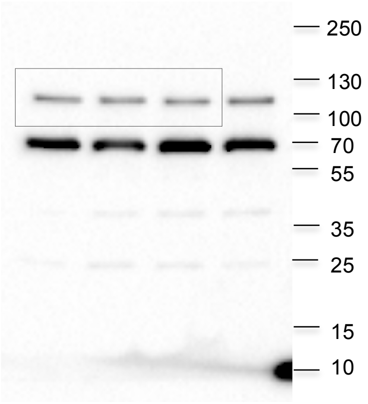

Fig. 3bVPS351% Input

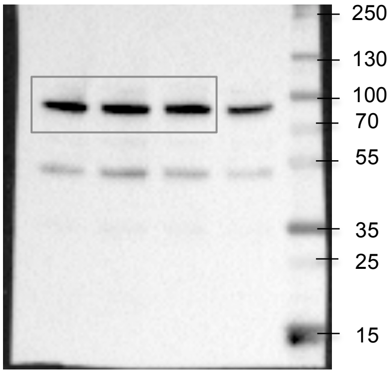

Fig. 3b IFNAR1 1% Input

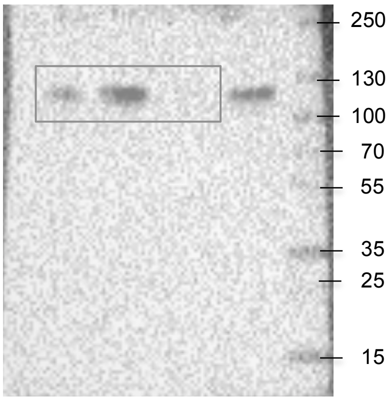

Fig. 3b IFNAR2 IP

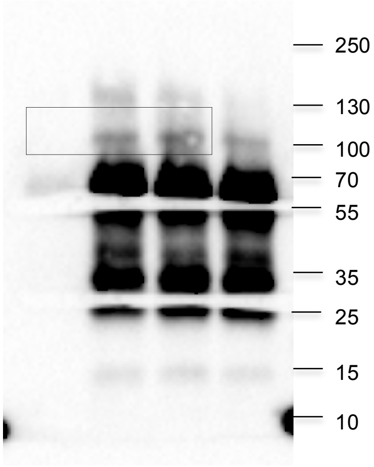

Fig. 3b VPS35 IP

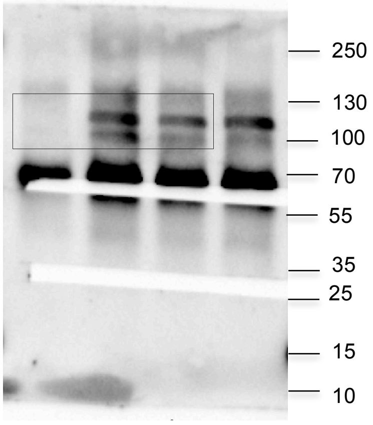

Fig. 4e CHC

Fig. 4e VPS35

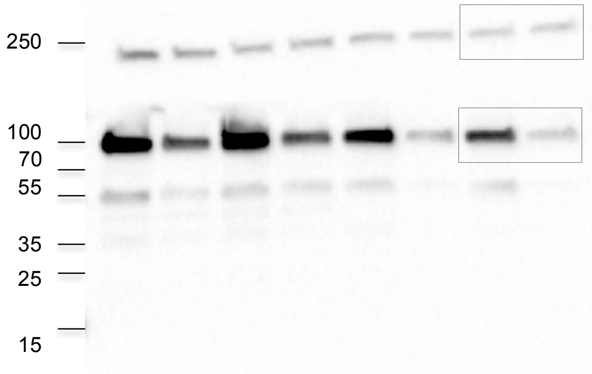

Supplementary Figure 7. continued

Fig. 6b SNAP

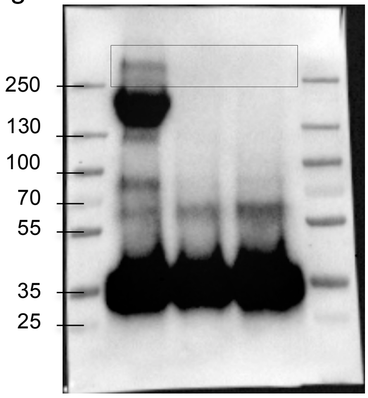

Fig. 7a pSTAT1 0min, 10 min, 40 min

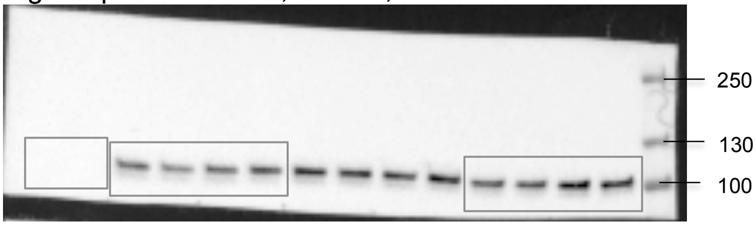

Fig. 7a pSTAT1 60 min

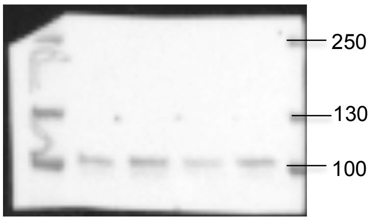

Fig. 7a STAT1 0 min, 10 min, 40 min

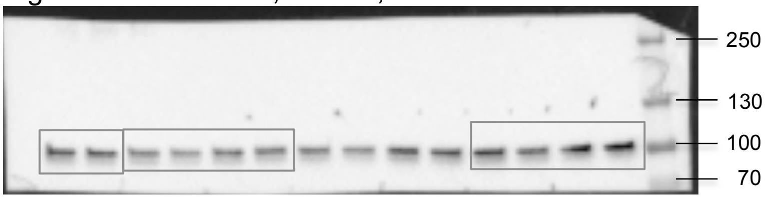

Fig. 7a STAT1 60 min

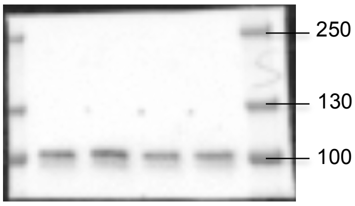

Fig. 7a tubulin 0min, 10 min, 40 min

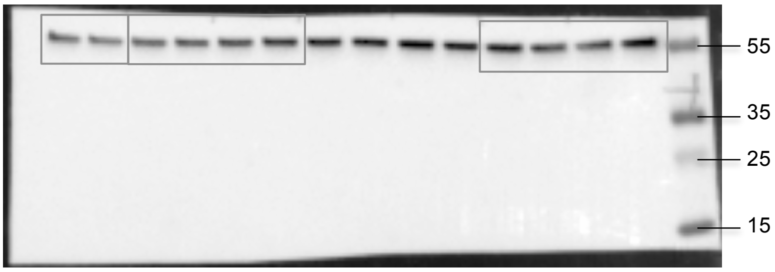

Fig. 7a tubulin 60 min

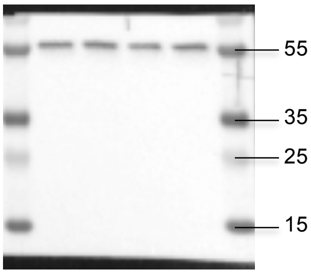

Supplementary Fig. 2a IFNAR1 1% Input

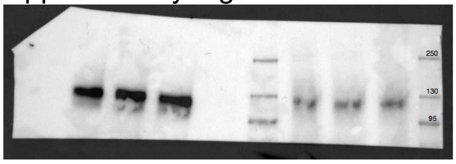

Supplementary Fig. 2a VPS35 1% Input

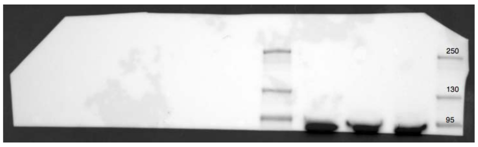

Supplementary Fig. 2a IFNAR1 IP

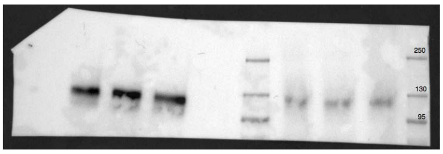

Supplementary Fig. 2a VPS35 IP

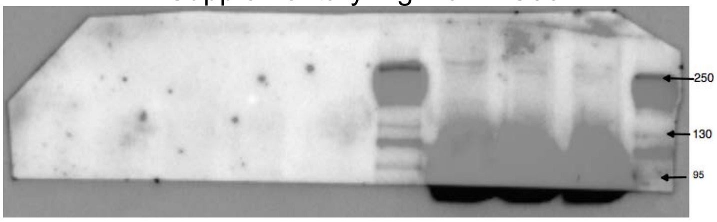

Supplementary Figure 7. continued

Supplementary Fig. 4a pSTAT1

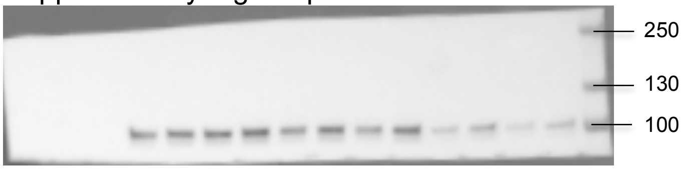

Supplementary Fig. 4a STAT1 0min, 10min, 40min, 80min IFNa

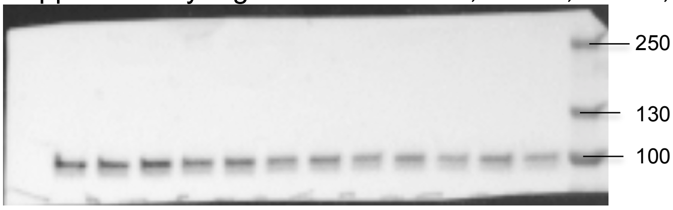

80min IFNb

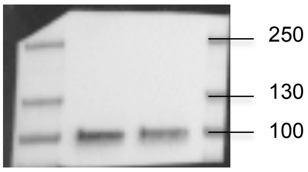

Supplementary Fig. 4a tubulin

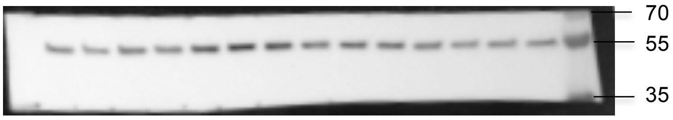

Supplementary Fig. 4a Rab7

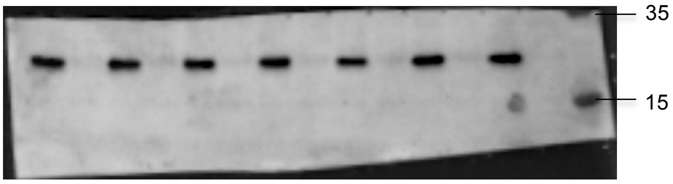

Supplementary Figure 7. continued

Selected proteins	Peptides								MW (kDa)	Description
	Beads		Ctrl		10 min IFNα		10 min IFNβ			
IFNAR2	**-**	*-*	**1**	*1*	**-**	-	**1**	*1*	57,8	Interferon alpha/beta receptor 2
STAT1	**-**	*-*	**3**	*2*	**1**	1	**4**	*1*	87,3	Signal transducer and activator of transcription 1-alpha/beta
CLH1	**13**	*6*	**21**	*6*	**4**	4	**12**	*3*	191,6	Clathrin heavy chain 1
AP2A1	**1**	*2*	**3**	*4*	**1**	1	**1**	*2*	107,5	AP-2 complex subunit alpha-1
AP2B1	**-**	*-*	**1**	*3*	**-**	-	**3**	*1*	104,6	AP-2 complex subunit beta
AP2M1	**-**	*-*	**2**	*1*	**-**	-	**-**	*-*	49,7	AP-2 complex subunit mu
EEA1	**1**	*2*	**3**	*1*	**-**	-	**1**	*1*	162,5	Early endosome antigen 1
RAB5A	**-**	*-*	**-**	*-*	**-**	-	**2**	*1*	23,7	Ras-related protein Rab-5A
RAB5C	**5**	*1*	**4**	*1*	**3**	1	**4**	*1*	23,5	Ras-related protein Rab-5C
RAB7A	**5**	*1*	**5**	*2*	**3**	1	**3**	*1*	23,5	Ras-related protein Rab-7a
RAB10	**1**	*1*	**2**	*1*	**1**	1	**1**	*1*	22,5	Ras-related protein Rab-10
RAB11A	**4**	*1*	**5**	*1*	**1**	*1*	**1**	*1*		Ras-related protein Rab-11A
RAB14	**4**	*1*	**5**	*1*	**2**	1	**2**	*1*	23,9	Ras-related protein Rab-14
RAB15	**1**	*2*	**1**	*3*	**1**	2	**1**	*1*	24,4	Ras-related protein Rab-15
RAB18	**3**	*1*	**7**	*1*	**1**	1	**4**	*1*	23,0	Ras-related protein Rab-18
RAB35	**1**	*2*	**2**	*1*	**1**	1	**1**	*1*	23,0	Ras-related protein Rab-35
VPS35	**1**	*1*	**5**	*2*	**4**	1	**4**	*1*	91,7	Vacuolar protein sorting-associated protein 35
VPS29	**-**	*-*	**2**	*1*	**1**	1	**-**	*-*	20,5	Vacuolar protein sorting-associated protein 29
VPS26A	**3**	*1*	**3**	*2*	**1**	*1*	**2**	*1*		Vacuolar protein sorting-associated protein 26A
SNX5	**-**	*-*	**1**	*1*	**-**	-	**1**	*1*	46,8	Sorting nexin-5
SNX2	**-**	*-*	**-**	*-*	**-**	-	**1**	*1*	58,5	Sorting nexin-2
SNX4	**-**	*-*	**1**	*1*	**-**	-	**-**	*-*	51,9	Sorting nexin-4
LAMP1	**2**	*4*	**3**	*1*	**2**	2	**3**	*1*	44,9	Lysosome-associated membrane glycoprotein 1
LAMP2	**2**	*3*	**2**	*5*	**1**	1	**2**	*2*	45,0	Lysosome-associated membrane glycoprotein 2
CD63	**3**	*3*	**4**	*2*	**2**	1	**3**	*1*	25,6	CD63 antigen

Supplementary Table 1. Chosen partners of endogenous IFNAR2 as analyzed by mass spectrometry. The numbers in **bold**: number of protein peptides found in the band with the highest concentration of the given protein peptides; numbers in *italics*: total number of bands in which peptides of the given protein were found.
